# The transcriptome of wild-type and immortalized corneal epithelial cells

**DOI:** 10.1038/s41597-021-00908-9

**Published:** 2021-05-07

**Authors:** Kai Furuya, Tao Wu, Ai Orimoto, Eriko Sugano, Hiroshi Tomita, Tohru Kiyono, Takahiro Kurose, Yoshihiro Takai, Tomokazu Fukuda

**Affiliations:** 1 Graduate School of Science and Engineering, Iwate University, 4-3-5 Ueda, Morioka, Iwate, 020-8551 Japan; 2 Exploratory Oncology Research and Clinical Trial Center, National Cancer Center, 6-5-1 Kashiwanoha, Kashiwa, Chiba, 277-8577 Japan; 3 Rohto Pharmaceutical Co., Ltd., Basic Research Development Division, 6-5-4 Kunimidai, Kizugawa, Kyoto, 619-0216 Japan

**Keywords:** RNA sequencing, Cell growth

## Abstract

Cellular immortalization enables indefinite expansion of cultured cells. However, the process of cell immortalization sometimes changes the original nature of primary cells. In this study, we performed expression profiling of poly A-tailed RNA from primary and immortalized corneal epithelial cells expressing Simian virus 40 large T antigen (SV40) or the combination of mutant cyclin-dependent kinase 4 (CDK4), cyclin D1, and telomere reverse transcriptase (TERT). Furthermore, we studied the expression profile of SV40 cells cultured in medium with or without serum. The profiling of whole expression pattern revealed that immortalized corneal epithelial cells with SV40 showed a distinct expression pattern from wild-type cells regardless of the presence or absence of serum, while corneal epithelial cells with combinatorial expression showed an expression pattern relatively closer to that of wild-type cells.

## Abstract (summary)

**Table:** 

Measurement(s)	Molecular Profiling
Technology Type(s)	RNA sequencing
Sample Characteristic - Organism	Homo sapiens

Machine-accessible metadata file describing the reported data: 10.6084/m9.figshare.14093807

## Background & Summary

Cultured cells may stop proliferating owing to cellular senescence^1^. Cell culture stress may induce the accumulation of cell cycle negative regulators such as p16^2^. Shortening of the telomere sequence is known to halt cell proliferation^3^. Immortalization is the process of inducing cell proliferation beyond these limitations. Cellular immortalization with the Simian virus 40 large T antigen (SV40) was commonly used^4^. The expression of SV40 causes degradation of p53 protein and bypasses the negative feedback of pRB. The loss of the function of the two tumor suppressor signaling proteins, p53 and pRB, efficiently facilitates cell proliferation^4,5^. However, alterations in cellular characteristics and chromosomal abnormalities are frequently reported in SV40-expressing cells^6^ possibly only a fraction of cells after crisis due to telomerase shortening is immortalized. The SV40-mediated genomic instability is also associated with the loss of the function of p53, which is a guardian of the genome in human carcinogenesis^7^.

In recent years, the expression of R24C-mutant cyclin-dependent kinase 4 (CDK4) and cyclin D1 and telomere reverse transcriptase (TERT) has been reported to efficiently induce immortalization of human cells^8^. The conserved amino acid sequences of CDK4 and cyclin D1 allow immortalization of various animal cell types^9–14^, indicating that the functions of these cell cycle regulators are evolutionarily conserved from reptiles to humans. Although the negative feedback of pRB is bypassed, the chromosomal condition remains relatively intact owing to the potent function of p53 in these cells. Based on the characteristics of mutant CDK4, cyclin D1, and TERT, the established cell line was named as K4DT. Although K4DT immortalized cells are more advantageous than oncogenic SV40 to preserve the original nature of primary cells, there is no functional evidence for this notion and limited data are available on epithelial-derived cells. For gene expression profiling, RNA-sequencing (RNA-seq) is a powerful method over the sequencing platform^15^. To evaluate biological characteristics, we carried out RNA-seq of conventional poly-A tailed RNA from human primary corneal epithelial cells and immortalized cells using the K4DT method or SV40.

## Methods

### Cell culture

We obtained human primary corneal epithelial cells from Lifeline Cell Technology (Lifeline, Frederick, MD, USA) through the local distributor Kurabo (Osaka, Japan). The primary cells were maintained in a life factor medium (Lifeline) as per the manufacturer’s instructions. The cells immortalized by the expression of R24C-mutant CDK4, cyclin D1, and TERT were described in our previous manuscript^16^. Based on the characteristics of the genes (CDK4, cyclin D1, and TERT) introduced, we named this immortalized cell line as K4DT. The corneal epithelial cells immortalized with the expression of SV40 (HCET) was obtained from Dr. Kaoru Araki-Sasaki (Osaka University, Osaka, Japan) through the RIKEN Cell Bank (RCB2280, Tsukuba, Japan). HCET was maintained in cell culture medium containing serum and in the life factor medium (Lifeline) after adaptation during three passages.

### RNA preparation and sequencing

Confluent primary and immortalized corneal epithelial cells were used for RNA extraction. The sampling (genomic DNA, protein extraction, cell cycle analysis, RNA extraction) from wild-type cells was carried out at passage 2. For K4DT cells, sampling (genomic DNA, protein extraction, cell cycle analysis, RNA extraction) was performed at passage 4. Sampling for SV40 cells (with and without serum) was completed at passage 4 after obtaining them from RIKEN Cell Bank.

For RNA extraction, the cells in 35 mm cell culture dishes were lysed in 700 μL RA1 solution from the NucleoSpin RNA extraction kit (code: 740955.250, Takara Bio, Shiga, Japan). We confirmed the quality of RNA using NanoDrop (ThermoFisher, Waltham, MA, USA), Qubit RNA Assay (ThermoFisher), and TapeStation (Agilent Technologies, Santa Clara, California, USA). RNA quality was confirmed to be more than 9.9 RIN value. In total, 500 ng of total RNA was used for library preparation with the NEB Next Ultra II RNA Directional Kit (New England Biolab, Ipswich, Massachusetts, USA). The quality of the library was evaluated using the Qubit DNA Assay (ThermoFisher) on TapeStation with D1000 screen Tape (Agilent Technologies). Triplicate samples for each group (wild-type, SV40 with serum, SV40 without serum, and K4DT) were processed for RNA-seq analysis. The cDNA samples were used for the sequencing reaction on the Illumina Hiseq X sequencing machine, resulting in approximately 29.4 to 48.6 M reads for each sample with 150 bp ends.

### Cell cycle analysis and western blotting

We carried out cell cycle analysis for wild-type, K4DT, SV40, and SV40 serum cells with the Muse Cell Cycle Assay Kit (cat. No. MCH100106, Merck Millipore, Billerica, MA, USA) using a cell analyzer (Merck Millipore). We obtained a protocol for fixation and analysis from the manufacturer and carried out western blotting to detect introduced proteins such as mutant CDK4, cyclin D, and SV40T. Primary antibodies against these proteins (anti-CDK4 [sc-56277, Santa Cruz Biotechnology, Dallas, TX, USA], anti-cyclin D1 [cat. No. 553, Medical & Biological Laboratories Co., Ltd., Nagoya, Japan], and anti-SV40 antibody [cat. No. sc-147, Santa Cruz Biotechnology]) were used for detection. The binding of primary antibodies to the target proteins was visualized with a goat anti-mouse IgG-labeled with horseradish peroxidase (HRP) (code no. 330, MBL) or goat anti-rabbit IgG labeled with HRP (code no. 458, MBL), Thermo Scientific Pierce ECL Substrate (ThermoFisher), and ImageQuant LAS-4000 Mini system (Fujifilm, Tokyo, Japan).

### RNA-seq data and downstream analysis

We checked the quality of the reads using the FastQC program. The adaptor sequence in the reads was removed using the PEAT program. After removal of the adaptor, the quality of reads was analyzed using FASTQC.

## Data Records

**Fig. 1:**
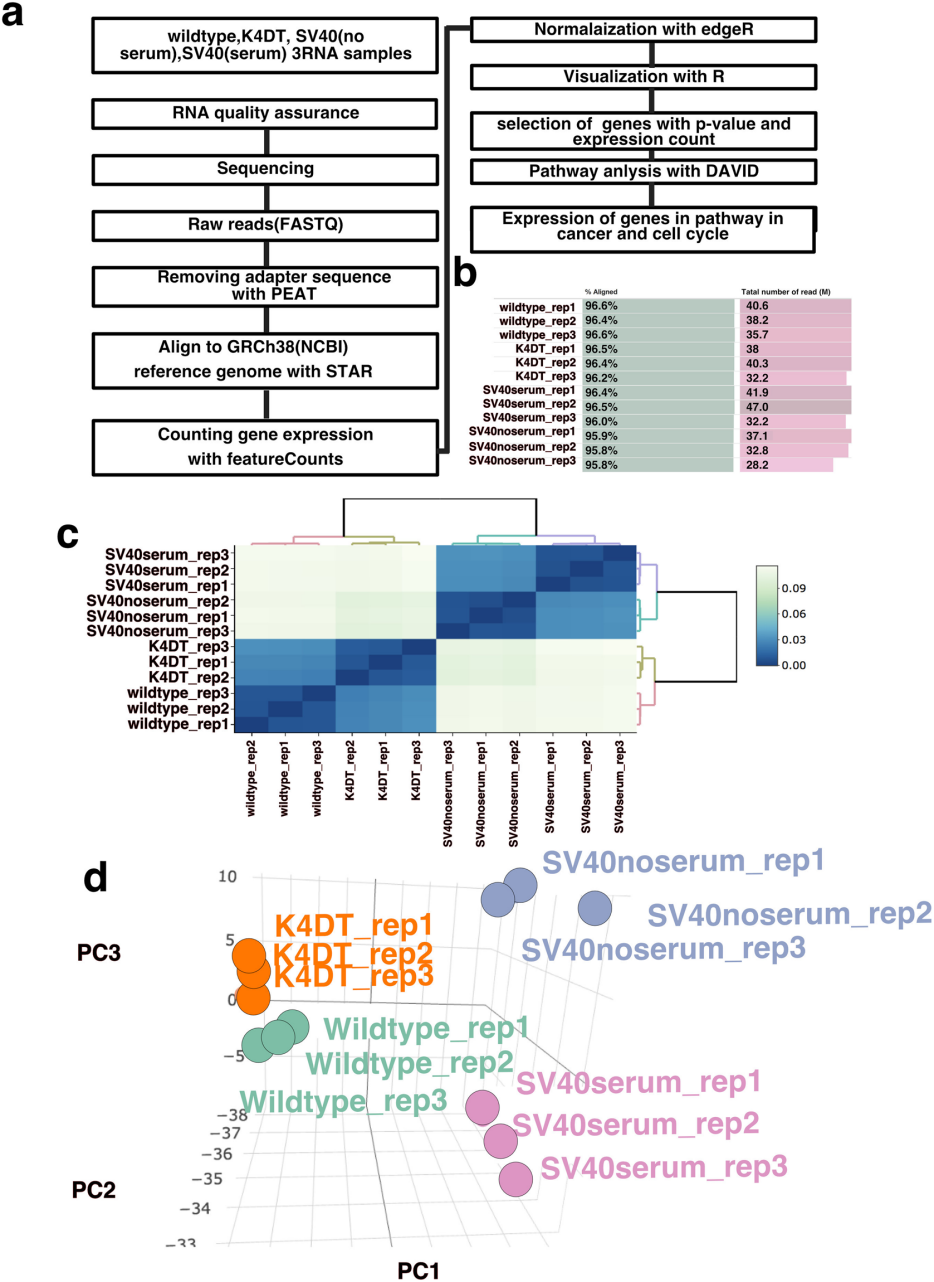
The workflow of RNA-seq of wild-type, K4DT, and SV40 immortalized corneal epithelial cells. (**a**), The workflow of RNA-seq. (**b**) Mapping ratio and total number of reads for each sample. (**c**) Correlation matrix plots of all samples. (**d**) Three-dimensional PCA of wild-type, K4DT, and SV40 cells.

The workflow of the RNA-seq analysis is shown in Fig. 1a. The sequencing data were paired-end with read lengths of 150 bp. The sequencing data were uploaded to Genbank through DDBJ (DNA Data Bank of Japan) with BioProject ID of PRJDB10909^17^. The mapping ratios of wild-type, K4DT, SV40 with serum, and SV40 without serum were approximately 95.8–96.6% (Fig. 1b). The quality of the reads is shown in Figures S2–S5. We obtained all data with biological triple replication. All average sequencing data lied within the green area, which is more than 26 and indicates their strong reliability. We carried out the mapping of reads using STAR. The BAM output files were processed with featureCount for the detection of expression counts. The expression counting data were further processed with TCC-GUI for downstream analysis. The complete list of gene expression has uploaded to Figshare^18^. Figure 1c shows the correlation matrix of the triplicated sequencing data. Each biological replicate formed unique clusters, indicating the reproducibility of the data. In Fig. 1d, we show the results of the three-dimensional principal component analysis (PCA). We also present the three-dimensional PCA details as a movie file (Movie S1)^19^. As shown in PCA results, the distance from wild-type to K4DT was smaller than that from wild-type to SV40 regardless of serum concentration.

We evaluated the differentially expressed (DE) genes after TMM normalization, and found 11925 genes (P < 0.01 at Edge R, and at least 300 counts in any sample). We uploaded the list of DE genes in Figshare^20^ and submitted it to the gene list for pathway analysis (DAVID 6.8). The first position listed in P-value was ribosome, second was ubiquitin-mediated proteolysis, third was endocytosis, and fourth was cell cycle. We selected the cell cycle pathway and ubiquitin-mediated proteolysis from the potential association with genomic instability.

**Fig. 2:**
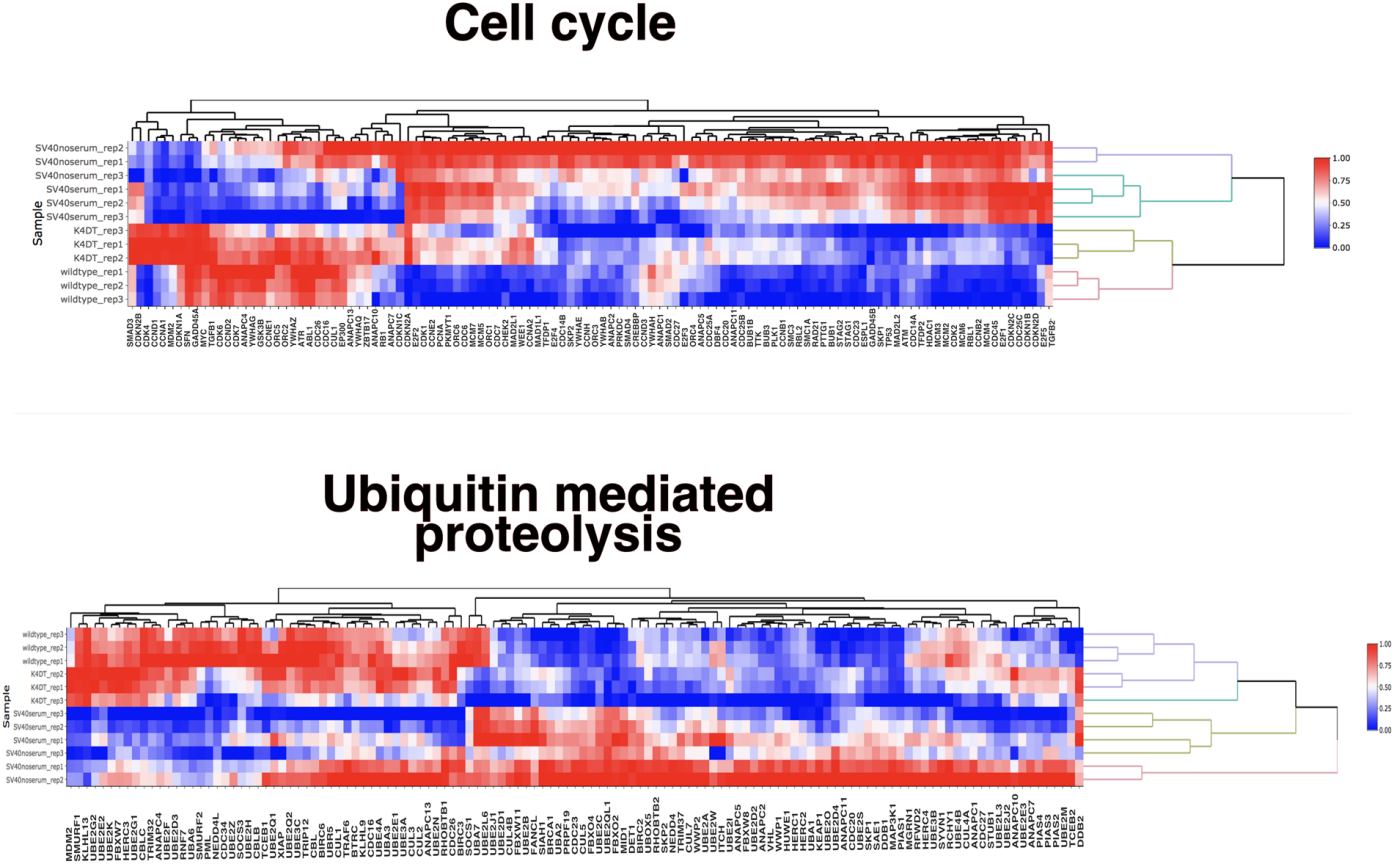
Heat map of cell cycle-related genes in the KEGG map and pathways related to cancer.

A heat map of the genes associated with cell cycle pathway and ubiquitin-mediated proteolysis is shown in Fig. 2. The distance from wild-type to K4DT was smaller than that from wild-type to SV40 within the cell cycle pathway and ubiquitin-mediated proteolysis.

**Fig. 3:**
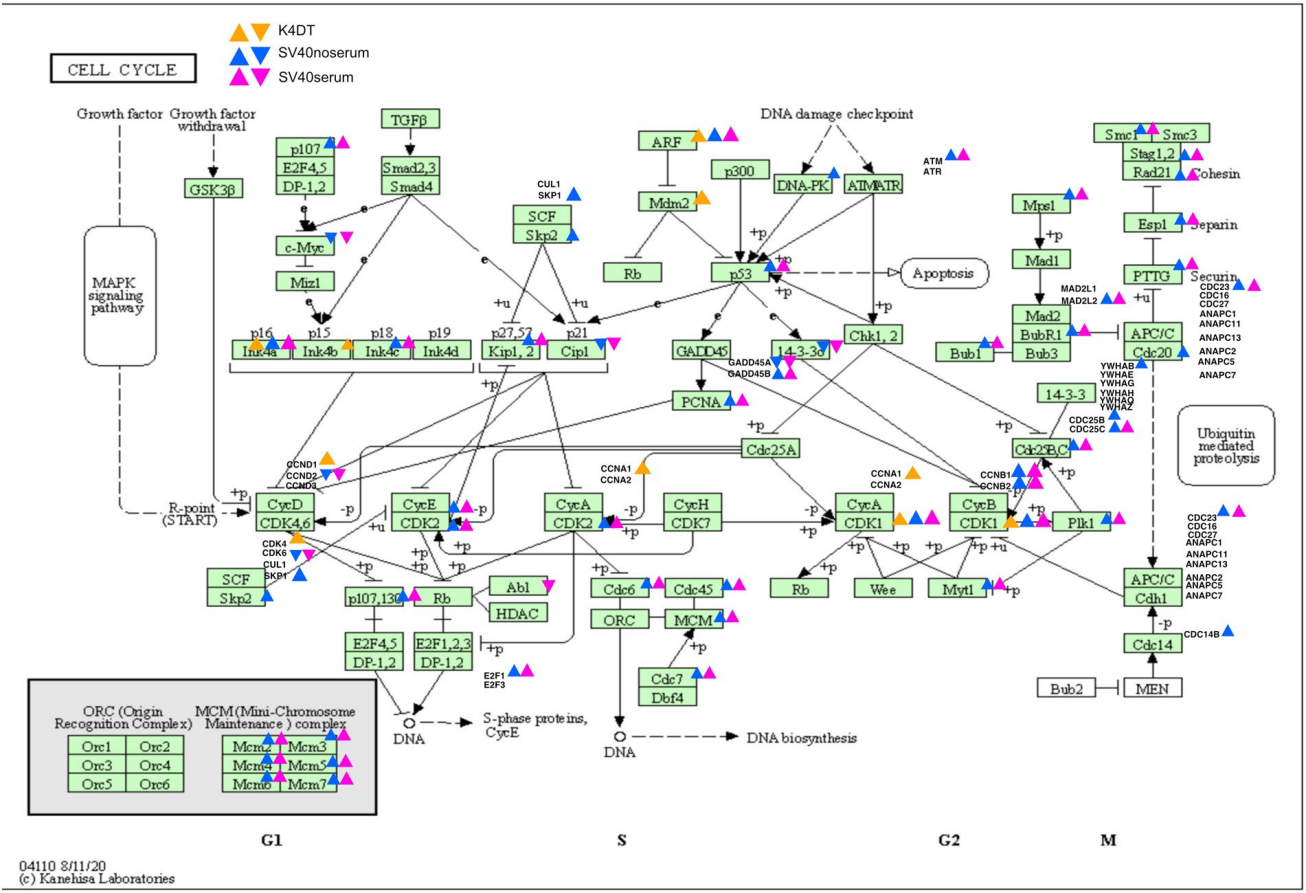
Mapping of upregulated or downregulated genes related to the cell cycle pathway. In bar plots, 2× upregulated or 1/2 downregulated genes were mapped in the KEGG pathway. Upper arrows indicate upregulated genes and lower arrows indicate downregulated genes in the corresponding sample.

**Fig. 4:**
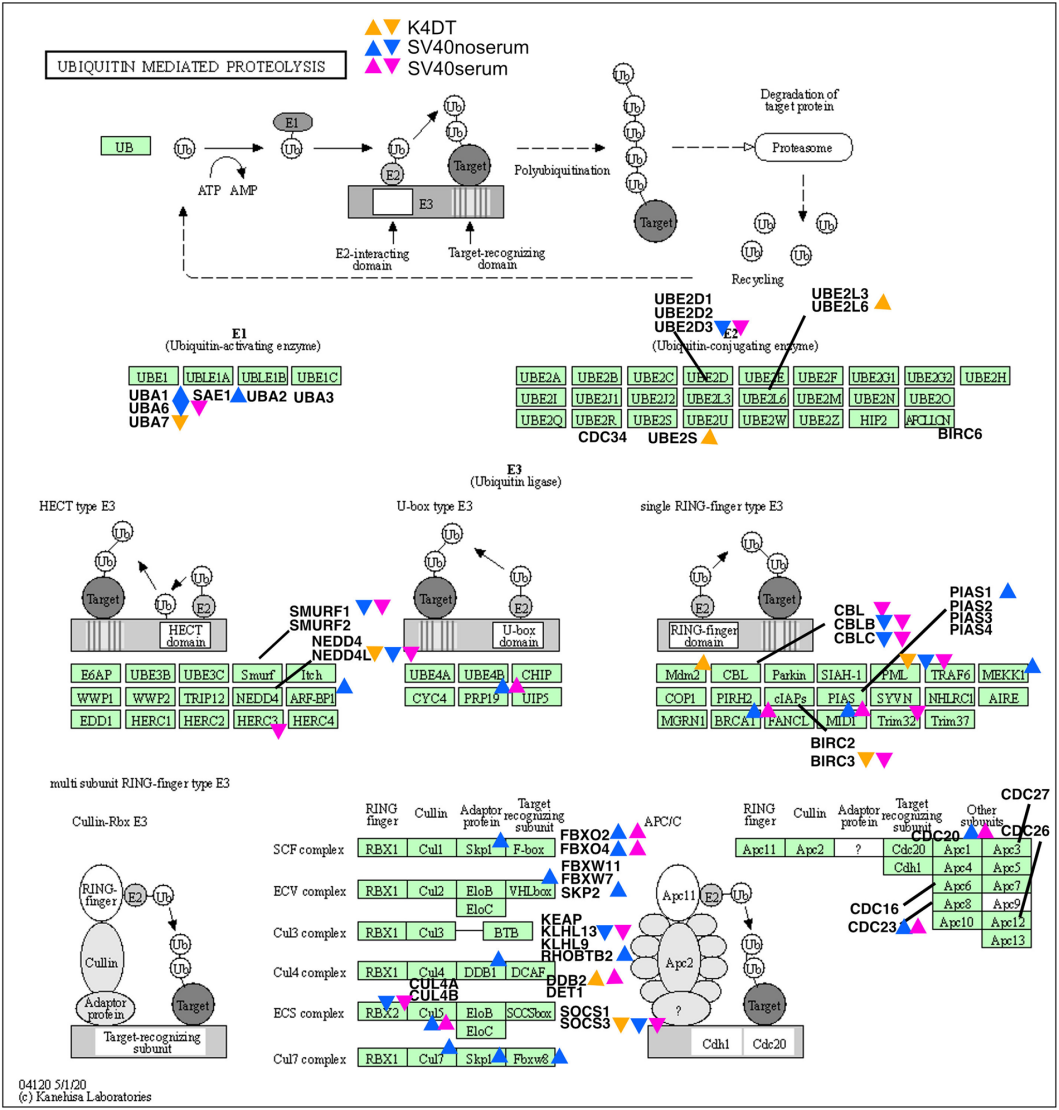
Mapping of upregulated or downregulated genes in the cell cycle pathway. In bar plots, 2× upregulated or 1/2 downregulated genes were mapped in the KEGG pathway. Upper arrows indicate upregulated genes and lower arrows indicate downregulated genes in the corresponding sample.

We next marked the genes that were more than two-fold upregulated or less than 0.5-fold downregulated in the Kyoto Encyclopedia for Genes and Genomes (KEGG) map of cell cycle (Fig. 3) and ubiquitin-mediated proteolysis (Fig. 4). In the cell cycle pathway, K4DT cells showed upregulated genes around the p16-pRB pathway, while SV40 cells showed upregulated genes throughout the cell cycle (Fig. 3). In ubiquitin-mediated proteolysis, the F-box-related molecules of the Skp, Cullin, F-box containing complex (SCF) complex, such as FBXO2, FBXO4, and SKP2, were upregulated in SV40 immortalized cells. From these data, we conclude that the expression difference from wild-type to K4DT cells was smaller than that from wild-type to SV40 cells regardless of the presence or absence of serum.

## Technical Validation

**Fig. 5:**
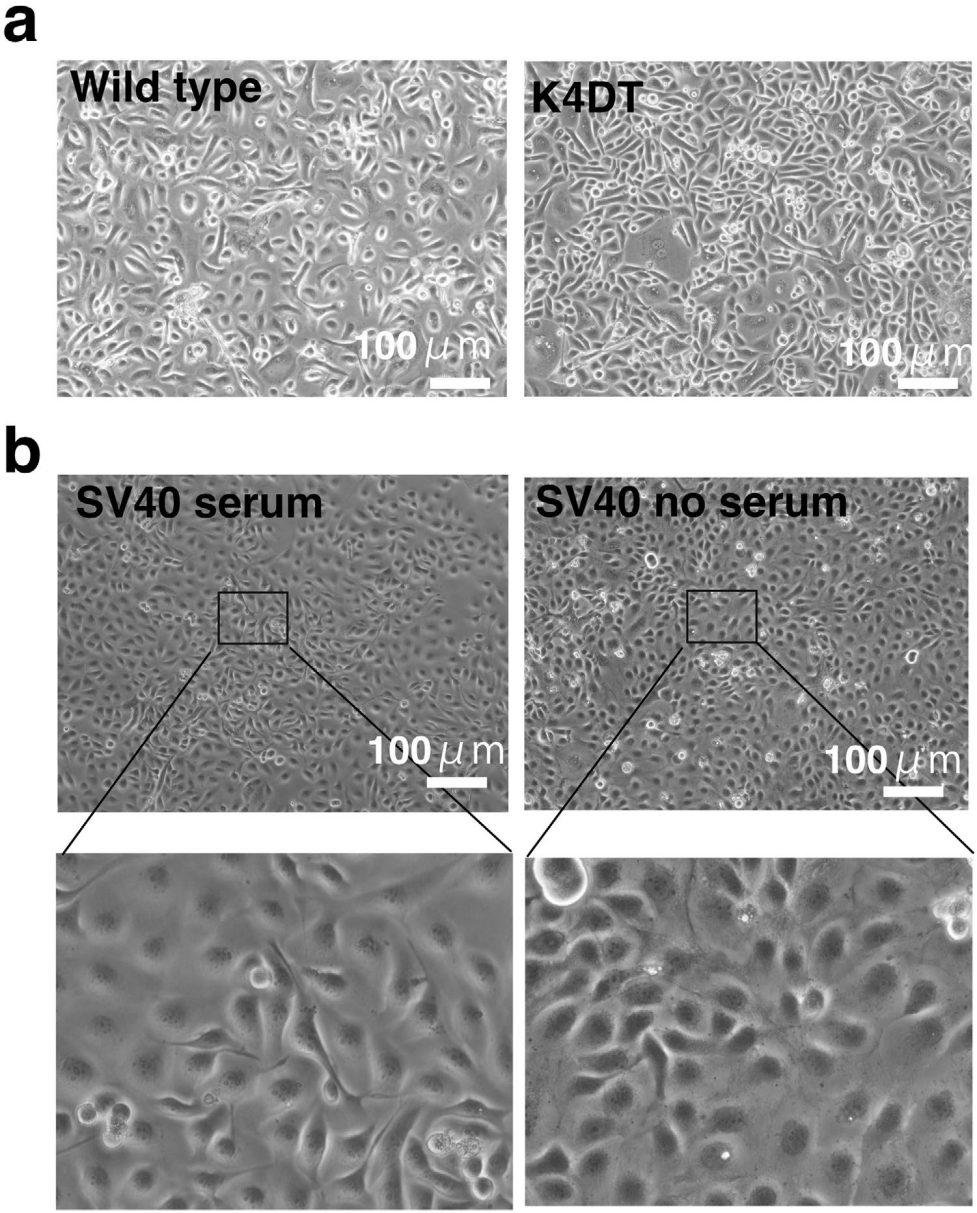
Morphologies of wild-type, K4DT, and SV40 immortalized corneal epithelial cells. (**a**) Wild-type (left panel) and K4DT immortalized corneal epithelial cells (right panel). (**b**) Morphology of SV40 immortalized corneal epithelial cells with serum (left panels) and without serum (right panels).

**Fig. 6:**
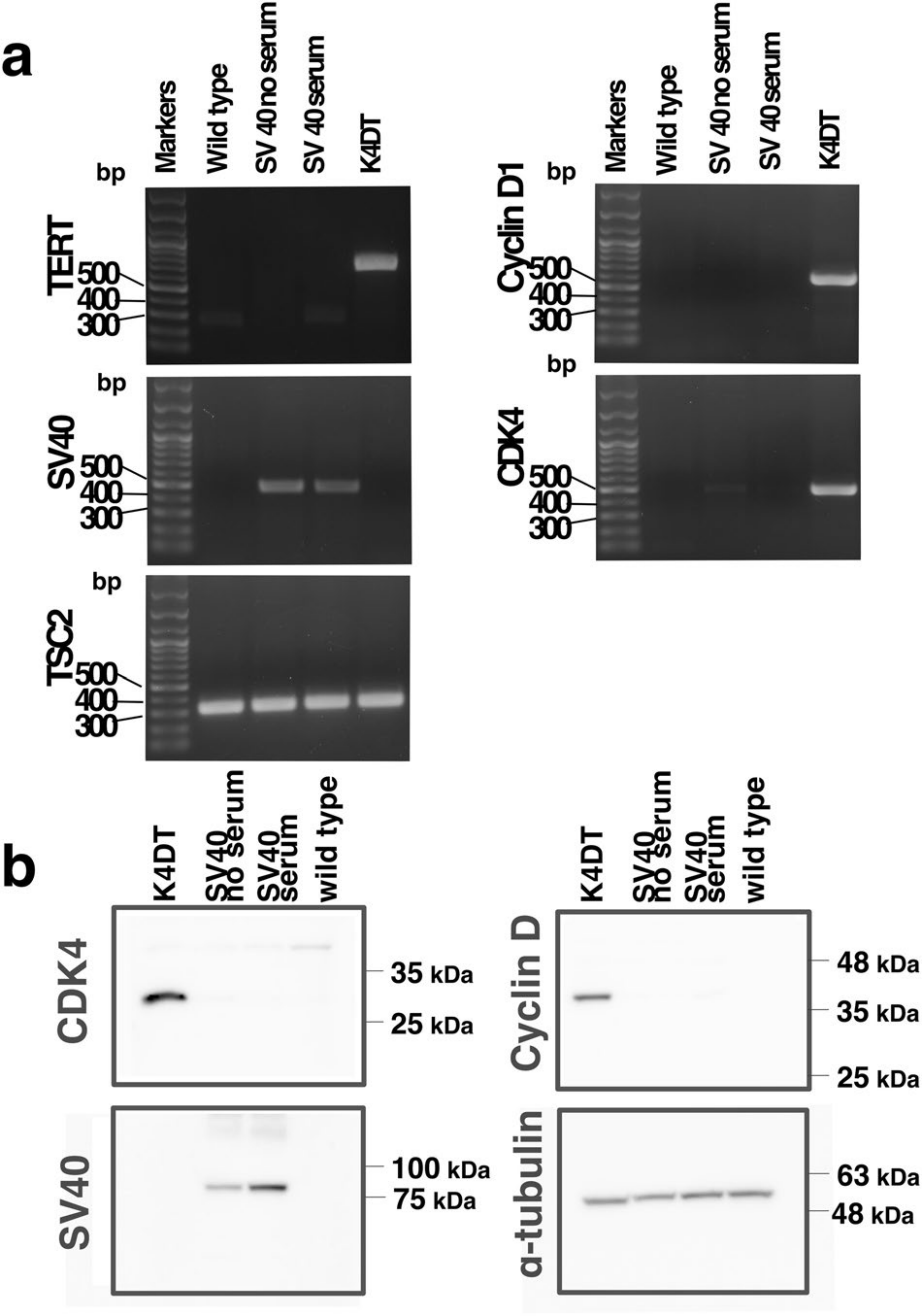
PCR genotyping and detection of gene products by western blotting. (**a**) PCR products amplified from *CDK4*, *cyclin D1*, *SV40*, *TERT*, and endogenous *TSC2* genes are listed. (**b**) Western blot analysis of anti-CDK4, SV40, cyclin D1.

**Fig. 7:**
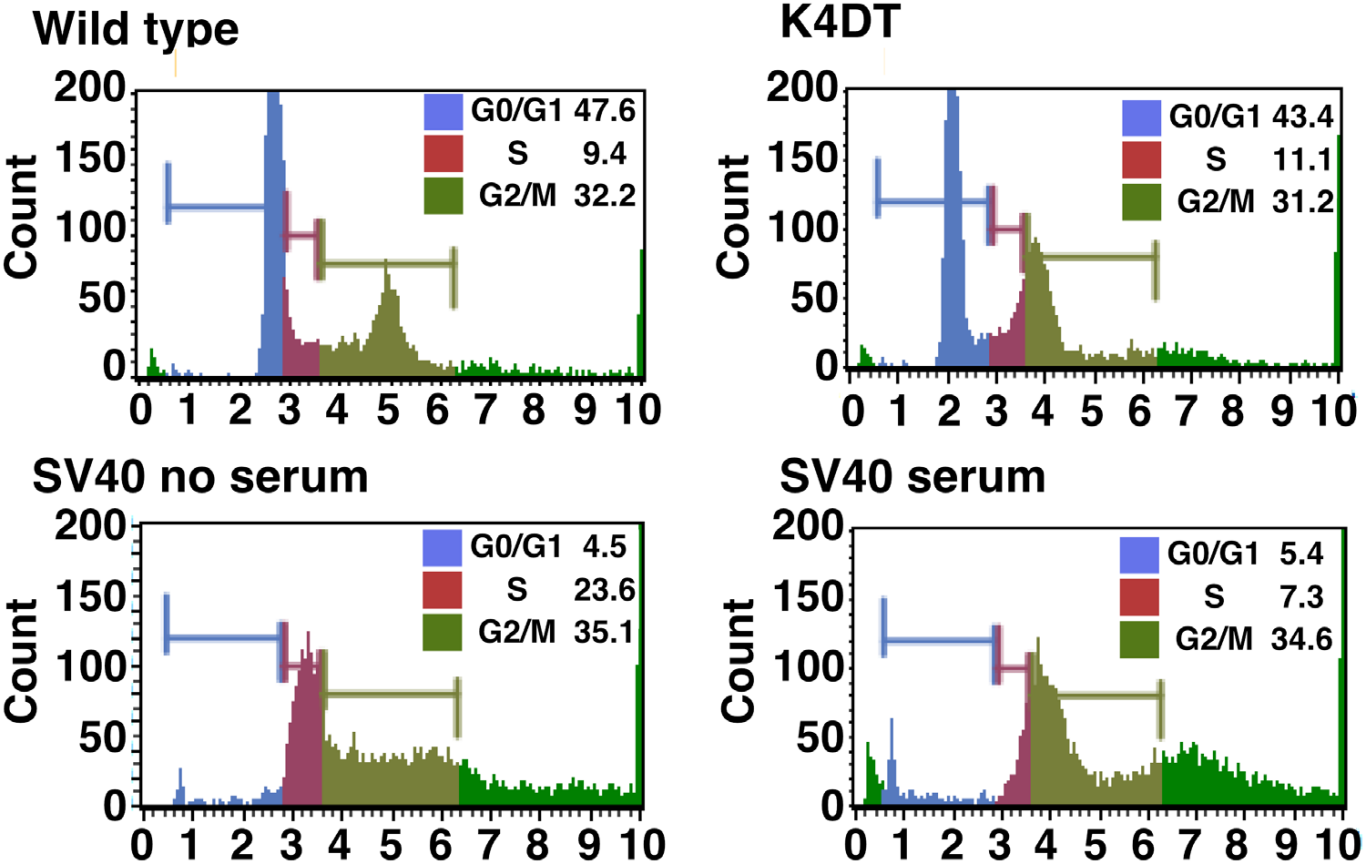
Analysis of cycle progression in wild-type, K4DT, and SV40 immortalized corneal epithelial cells. Representative results were shown.

We used wild type cell derived cell used as negative control for immortalized cell, and we used SV40T immortalized cell with serum condition (recommended cell culture condition from RIKEN cell bank) as positive control cell for immortalization. We listed the morphology of wild-type human-derived corneal epithelial cells (Fig. 5a, left side) and K4DT cells (Fig. 5a, right side). The immortalized human-derived corneal epithelial cells expressing SV40 were maintained in a cell culture medium supplemented with serum (Fig. 5b, left side) or in a medium without serum (Fig. 5b, right side). We confirmed that the growth of SV40 cells was not affected in the absence of serum during the sequential passage experiments (Figure S1). PCR analysis of the genomic DNA from cells (Fig. 6a and Figure S23) showed good agreement with the predicted combination of exogenously introduced transgenes. Cell cycle analysis results showed a diploid pattern in wild-type and K4DT cells, while SV40 cells showed a broad signal of more than 4 N suggestive of polyploid formation (Fig. 7). In brief, in wild type and K4DT cell, we can observe the two peaks of histogram (higher peak is 2n,lower peak is 4n). However, regardless of presence and absence of serum, SV40 cell showed mobility shift to left side, and first peak was observed around 4n and broad signals in left side of the peak. We also detected the expression of the proteins encoded by the transduced genes by western blotting (Fig. 6b and Figure S24). We listed the expression levels of cell cycle-related genes in the bar plots of Figures S6–S13. In addition, we showed the expression levels of ubiquitin-mediated proteolysis-related genes in bar plots of Figures S14–S21. We furthermore detected the chromosome number per cell with Giemsa staining using 50 mitotic cells. While K4DT showed 49 cell maintained normal 46 chromosome, SV40 cell showed broad peak around 63 chromosome, indicating the chromosome instability (Figure S22a). Furthermore, we showed representative G-banding pattern of K4DT and SV40 cell (Figure S22b). While K4DT keeps diploid condition, SV40 cell showed polyploid condition with intensive chromosome abnormalities.

## Supplementary information

Supplementary Material

## Data Availability

We listed the names and versions of the softwares used for data analysis. FastQC, version 0.11.3, was used for quality check of the raw FASTQ sequencing file. https://www.bioinformatics.babraham.ac.uk/projects/fastqc/. PRINSEQ, version 0.20.4, was used to remove low-quality reads. http://prinseq.sourceforge.net/. PEAT, version 1.2, was used to remove the adaptor sequence. https://github.com/jhhung/PEAT. STAR, version 2.6.1 was used for mapping. https://github.com/alexdobin/STAR. featureCount, SUBREAD, release 1.6.5 was used for the expression counting. http://subread.sourceforge.net. R package, version 4.0.3, was used for the downstream analysis. https://www.r-project.org. TCC-GUI tool was used for the downstream analysis. https://github.com/swsoyee/TCC-GUI.
