# Supplementary material for: The transcriptome of wild-type and immortalized corneal epithelial cells
Source: Sci Data. 2021 May 7;8:126. doi: 10.1038/s41597-021-00908-9 (PMC8105388; doi:10.1038/s41597-021-00908-9)
Supplement: Supplementary file 1 — Supplementary Material [file 41597_2021_908_MOESM1_ESM.pdf]

Title

The transcriptome of wild-type and immortalized corneal epithelial cells

Authors

Kai Furuya[1], Tao Wu[1], Ai Orimoto[1], Eriko Sugano[1], Hiroshi Tomita[1], Tohru Kiyono[2*], Takahiro Kurose[3], Yoshihiro Takai[3*], Tomokazu Fukuda[1*]

Affiliations

[1]Graduate School of Science and Engineering, Iwate University, 4-3-5 Ueda, Morioka, Iwate 020-8551, Japan

[2]Exploratory Oncology Research and Clinical Trial Center, National Cancer Center, 6-5-1 Kashiwanoha, Kashiwa, Chiba 277-8577, Japan

[3]Rohto Pharmaceutical Co., Ltd., Basic Research Development Division, 6-5-4 Sumidai, Kizugawa, Kyoto 619-0216, Japan

*Corresponding Author: Tomokazu Fukuda (tomofukuda009@gmail.com), Tohru Kiyono (tkiyono@ncc.go.jp), Yoshihiro Takai (takai@rohto.co.jp)

Figure S1. Population doubling of SV40 immortalized corneal epithelial cells without serum or with serum. The mean values of duplicated samples were plotted. We did not detect any obvious difference in cellular morphology between serum and no serum conditions. Page 1

Figure S2. FASTQC quality check of reads obtained from wild-type corneal epithelial cells. Page 2

Figure S3. FASTQC quality check of reads obtained from K4DT immortalized corneal epithelial cells. Page 3

Figure S4. FASTQC quality check of reads obtained from SV40 immortalized corneal epithelial cells, which were maintained in cell culture medium without serum. Page 4

Figure S5. FASTQC quality check of reads obtained from SV40 immortalized corneal epithelial cells, which were maintained in cell culture medium with serum. Page 5

Figure S6. Bar plots of cell cycle-related genes in wild-type, K4DT immortalized, and SV40 immortalized corneal epithelial cells maintained in cell culture medium with or without serum. The expression levels of *ABL1*, *ANAPC1*, *ANAPC10*, *ANAPC11*, *ANAPC13*, *ANAPC2*, *ANAPC4*, *ANAPC5*, *ANAPC7*, ATM, *ATR*, *BUB1*, *BUB1B*, *BUB3*, *CCNA1*, and *CCNA2* are shown. Page 6

Figure S7. Bar plots of cell cycle-related genes in wild-type, K4DT immortalized, and SV40 immortalized corneal epithelial cells maintained in cell culture medium with or without serum. The expression levels of *CCNB1*, *CCNB2*, *CCND1*, *CCND2*, *ARPC5*, *VVNE1*, *CCNE2*, *CCNH*, *CDC14A*, *CDC14B*, *CDC16*, *CDC20*, *CDC23*, *CDC25A*, *CDC25B*, and *CDC25C* are shown. Page 7

Figure S8. Bar plots of cell cycle-related genes in wild-type, K4DT immortalized, and SV40 immortalized corneal epithelial cells maintained in cell culture medium with or without serum. The expression levels of *CDC26*, *CDC27*, *CDC45*, *CDC6*, *CDC7*, *CDK1*, *CDK2*, *CDK4*, *CDK6*, *CDK7*, *CDKN1A*, *CDKN1B*, *CDKN1C*, *CDKN2A*, *CDKN2B*, and *CDKN2C* are shown. Page 8

Figure S9. Bar plots of cell cycle-related genes in wild-type, K4DT immortalized, and SV40 immortalized corneal epithelial cells maintained in cell culture medium with or without serum. The expression levels of *CDKN2D*, *CHEK2*, *CREBBP*, *CUL1*, *DBF4*, *E2F1*, *E2F2*, *E2F3*, *E2F4*, *E2F5*, *EP300*, *ESPL1*, *GADD45*, *GADD45B*, *GSK3B*, and *HDAC1* are shown. Page 9

Figure S10. Bar plots of cell cycle-related genes in wild-type, K4DT immortalized, and SV40 immortalized corneal epithelial cells maintained in cell culture medium with or without serum. The expression levels of *MAD1L1*, *MAD2L1*, *MAD2L2*, *MCM2*, *MCM3*, *MCM4*, *MCM5*, *MCM6*, *MCM7*, *MDM2*, *MYC*, *ORC1*, *ORC2*, *ORC3*, *ORC4*, and *ORC5* are shown. Page 10

Figure S11. Bar plots of cell cycle-related genes in wild-type, K4DT immortalized, and SV40 immortalized corneal epithelial cells maintained in cell culture medium with or without serum. The expression levels of *ORC6*, *PCNA*, *PKMYT1*, *PLK1*, *PRKDC*, *PTTG1*, *RAD21*, *RB1*, *RBL1*, *RBL2*, *SFN*, *SKP1*, *SKP2*, *SMAD2*, *SMAD3*, and *SMAD4* are shown. Page 11

Figure S12. Bar plots of cell cycle-related genes in wild-type, K4DT immortalized, SV40 immortalized corneal epithelial cell maintained in cell culture medium with or without serum. The expression levels of *SMC1A*, *SMC3*, *STAG1*, *STAG2*, *TFDP1*, *TFDP2*, *TGFB1*, *TGFB2*, *TP53*, *TTK*, *WEE1*, *YWHAB*, *YWHAE*, *YWHAG*, *YWHAH*, and *YWHAQ* are shown. Page 12

Figure S13. Bar plots of cell cycle-related genes in wild-type, K4DT immortalized, and SV40 immortalized corneal epithelial cells maintained in cell culture medium with or without serum. The expression levels of *YWHAZ* and *ZBTB17* are shown. Page 13

Figure S14. Bar plots of ubiquitin-mediated proteolysis-related genes in wild-type, K4DT immortalized, and SV40 immortalized corneal epithelial cells maintained in cell culture medium with or without serum. The expression levels of *ANAPC1, ANAPC10, ANAPC11, ANAPC13, ANAPC2, ANAPC4, ANAPC5, ANAPC7, BIRC2, BIRC3, BIRC6, BRCA1, BTRC, CBL, CBLB, CBLC* are shown. Page 13

Figure S15. Bar plots of ubiquitin-mediated proteolysis-related genes in wild-type, K4DT immortalized, and SV40 immortalized corneal epithelial cells maintained in cell culture medium with or without serum. The expression levels of *CDC16*, *CDC20*, *CDC23*, *CDC26*, *CDC27*, *CDC34*, *CUL1*, *CUL2*, *CUL3*, *CUL4A*, *CUL4B*, *CUL5*, *CUL7*, *DDB1*, *DDB2*, and *DET1* are shown. Page 15

Figure S16. Bar plots of ubiquitin-mediated proteolysis-related genes in wild-type, K4DT immortalized, and SV40 immortalized corneal epithelial cells maintained in cell culture medium with or without serum. The expression levels of *FANCL*, *FBXO2*, *FBXO4*, *FBXW11*, *FBXW7*,

FBXW8, *HERC1*, *HERC2*, *HERC3*, *HERC4*, *HUWE1*, *ITCH*, *KEAP1*, *KLHL13*, *KLHL9*, and *MAP3K1* are shown. Page 16

Figure S17. Bar plots of ubiquitin-mediated proteolysis-related genes in wild-type, K4DT immortalized, and SV40 immortalized corneal epithelial cells maintained in cell culture medium with or without serum. The expression levels of *MDM2*, *MGRN1*, *MID1*, *NEDD4*, *NEDD4L*, *PIAS1*, *PIAS2*, *PIAS3*, *PIAS4*, *PML*, *PRPF19*, *RCHY1*, *RFWD2*, *RHOBTB1*, *RHOBTB2*, and *RNF7* are shown. Page 17

Figure S18. Bar plots of ubiquitin-mediated proteolysis-related genes in wild-type, K4DT immortalized, and SV40 immortalized corneal epithelial cells maintained in cell culture medium with or without serum. The expression level of *SAE1*, *SIAH1*, *SKP1*, *SKP2*, *SMURF1*, *SMURF2*, *SOCS1*, *SOCS3*, *STUB1*, *SYVN1*, *TCEB1*, *TCEB2*, *TRAF6*, *TRIM32*, *TRIM37*, and *TRIP12* are shown. Page 18

Figure S19. Bar plots of ubiquitin-mediated proteolysis-related genes in wild-type, K4DT immortalized, and SV40 immortalized corneal epithelial cells maintained in cell culture medium with or without serum. The expression levels of *UBA1*, *UBA2*, *UBA3*, *UBA6*, *UBA7*, *UBE2A*, *UBE2B*, *UBE2C*, *UBE2D1*, *UBE2D2*, *UBE2D3*, *UBE2D4*, *UBE2E1*, *UBE2E2*, *UBE2E3*, and *UBE2F* are shown. Page 19

Figure S20. Bar plots of ubiquitin-mediated proteolysis-related genes in wild-type, K4DT immortalized, and SV40 immortalized corneal epithelial cells maintained in cell culture medium with or without serum. *UBE2G1*, *UBE2G2*, *UBE2H*, *UBE2I*, *UBE2J1*, *UBE2J2*, *UBE2K*, *UBE2L3*, *UBE2L6*, *UBE2M*, *UBE2N*, *UBE2O*, *UBE2Q1*, *UBE2Q2*, *UBE2QL1*, and *UBE2S* expression levels are shown. Page 20

Figure S21. Bar plots of ubiquitin-mediated proteolysis-related genes in wild-type, K4DT immortalized, and SV40 immortalized corneal epithelial cells maintained in cell culture medium with or without serum. The expression levels of *UBE2W*, *UBE2Z*, *UBE3A*, *UBE3B*, *UBE3C*, *UBE4A*, *UBE4B*, *UBOX5*, *UBR5*, *VHL*, *WWP1*, *WWP2*, and *XIAP* are shown. Page 21

Figure S22. Chromosome analysis of corneal epithelial cells with K4DT and SV40T. (a) Number of chromosome of corneal epithelial cell with K4DT and SV40T. Although K4DT cell showed 49 mitotic cell with 46 chromosome, 24 mitotic SV40T cell showed 63 chromosomes. (b) Representative chromosome analysis of K4DT cell and SV40T cell. While K4DT cell

maintained diploid, SV40T showed intensive chromosome abnormalities with trisomy and existence of abnormal chromosomes (mar). Arrows indicates the positions of abnormalities. Page 22

Figure S23. Full-length photograph of PCR diagnosis gel shown in Figure 6a. Page 23

Figure S24. Full-length images western blots shown in Figure 6b. Page 24

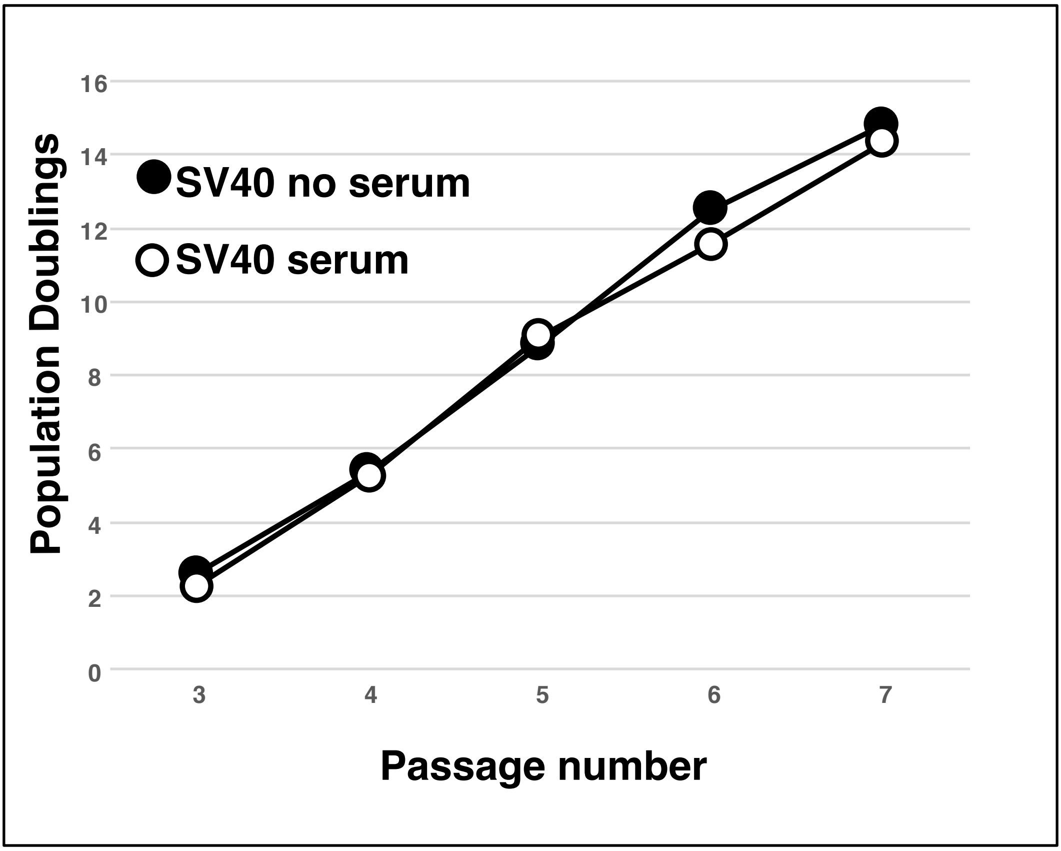
SV40 no serum
SV40 serum
Population Doublings
16
14
12
10
8
6
4
2
0
3
4
5
6
7
Passage number

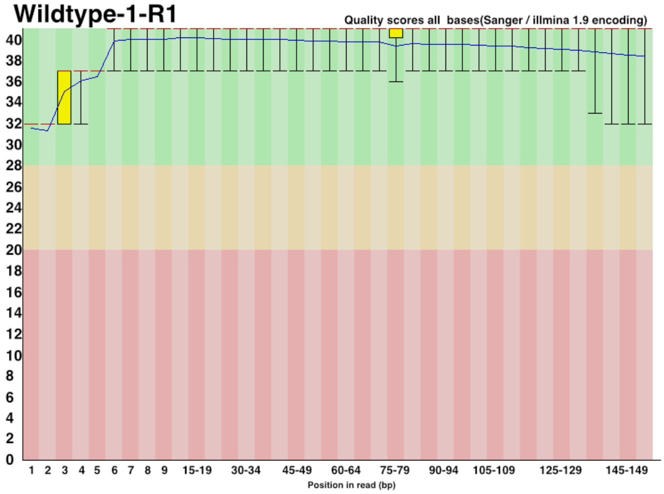
Wildtype-1-R1
Quality scores all bases(Sanger / illmina 1.9 encoding)
Position in read (bp)

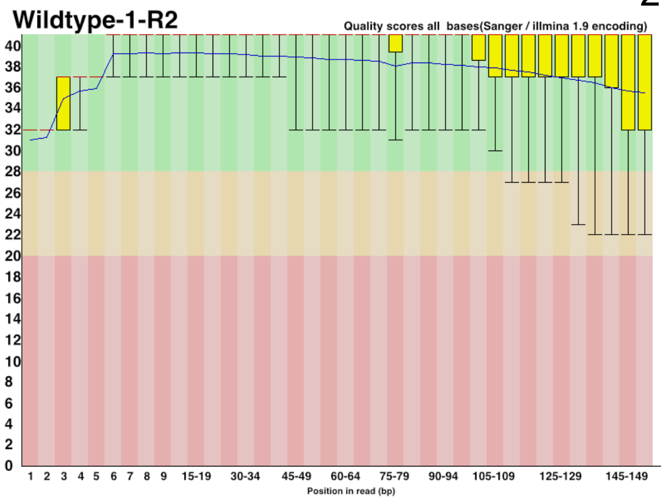
Wildtype-1-R2
Quality scores all bases(Sanger / illmina 1.9 encoding)
Position in read (bp)

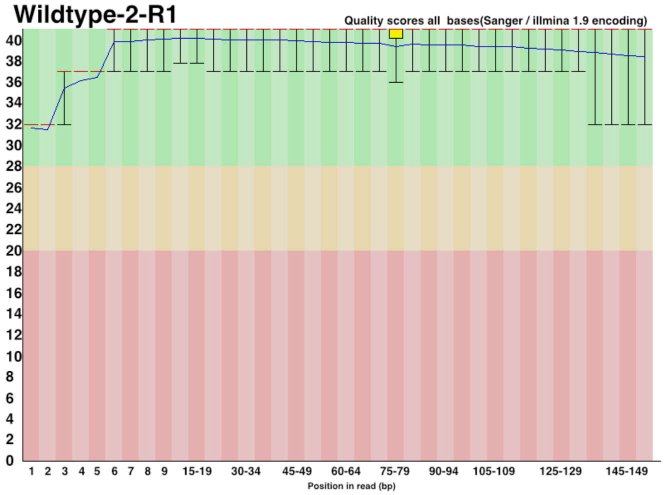
Wildtype-2-R1
Quality scores all bases(Sanger / illmina 1.9 encoding)
Position in read (bp)

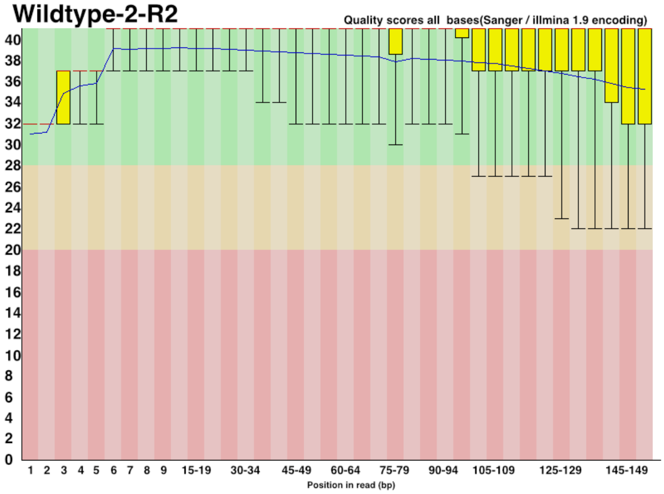
Wildtype-2-R2
Quality scores all bases(Sanger / illmina 1.9 encoding)
Position in read (bp)

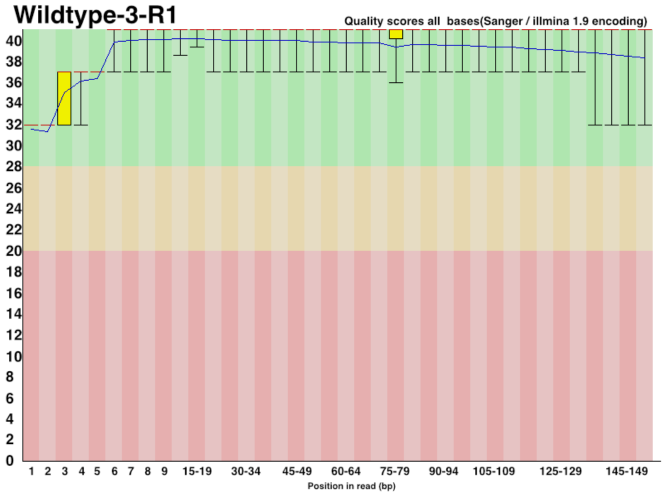
Wildtype-3-R1
Quality scores all bases(Sanger / illmina 1.9 encoding)
Position in read (bp)

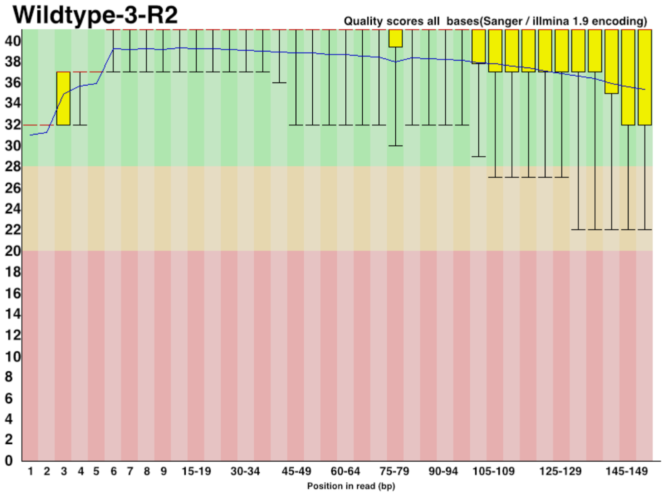
Wildtype-3-R2
Quality scores all bases(Sanger / illmina 1.9 encoding)
Position in read (bp)

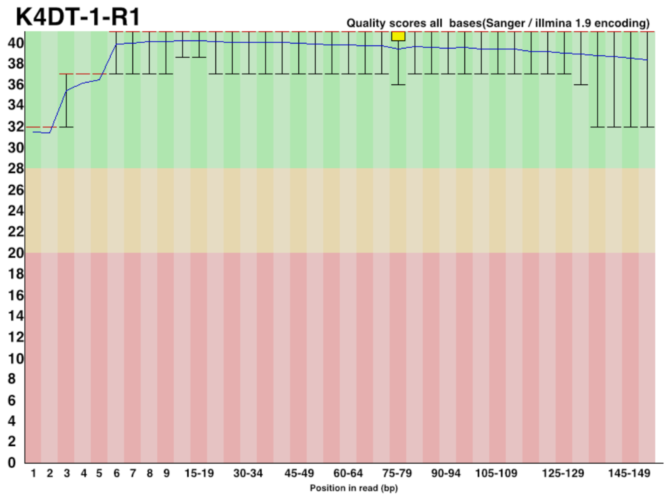
K4DT-1-R1
Quality scores all bases(Sanger / illmina 1.9 encoding)
40 38 36 34 32 30 28 26 24 22 20 18 16 14 12 10 8 6 4 2 0
1 2 3 4 5 6 7 8 9 15-19 30-34 45-49 60-64 75-79 90-94 105-109 125-129 145-149
Position in read (bp)

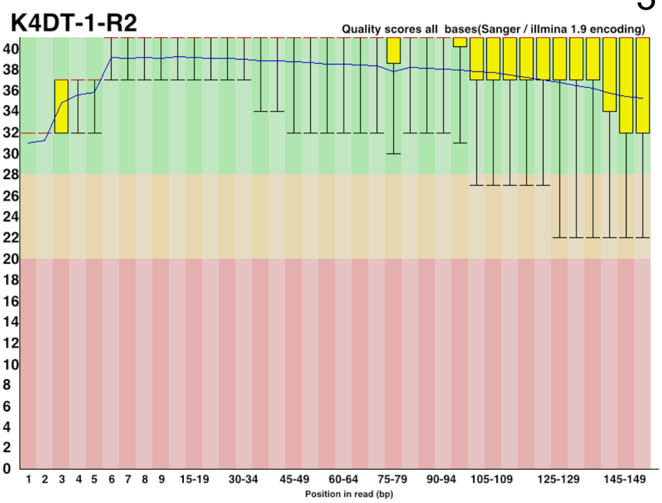
K4DT-1-R2
Quality scores all bases(Sanger / illmina 1.9 encoding)
40 38 36 34 32 30 28 26 24 22 20 18 16 14 12 10 8 6 4 2 0
1 2 3 4 5 6 7 8 9 15-19 30-34 45-49 60-64 75-79 90-94 105-109 125-129 145-149
Position in read (bp)

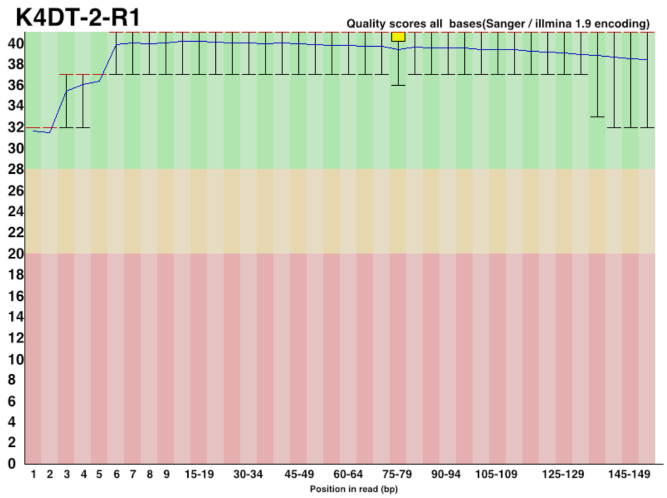
K4DT-2-R1
Quality scores all bases(Sanger / illmina 1.9 encoding)
40 38 36 34 32 30 28 26 24 22 20 18 16 14 12 10 8 6 4 2 0
1 2 3 4 5 6 7 8 9 15-19 30-34 45-49 60-64 75-79 90-94 105-109 125-129 145-149
Position in read (bp)

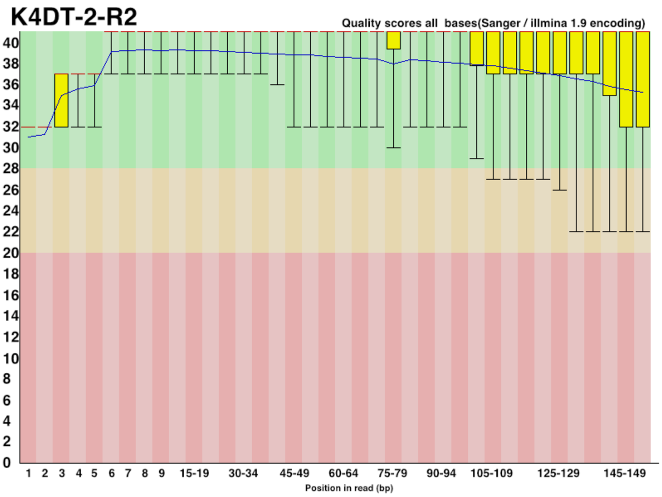
K4DT-2-R2
Quality scores all bases(Sanger / illmina 1.9 encoding)
40 38 36 34 32 30 28 26 24 22 20 18 16 14 12 10 8 6 4 2 0
1 2 3 4 5 6 7 8 9 15-19 30-34 45-49 60-64 75-79 90-94 105-109 125-129 145-149
Position in read (bp)

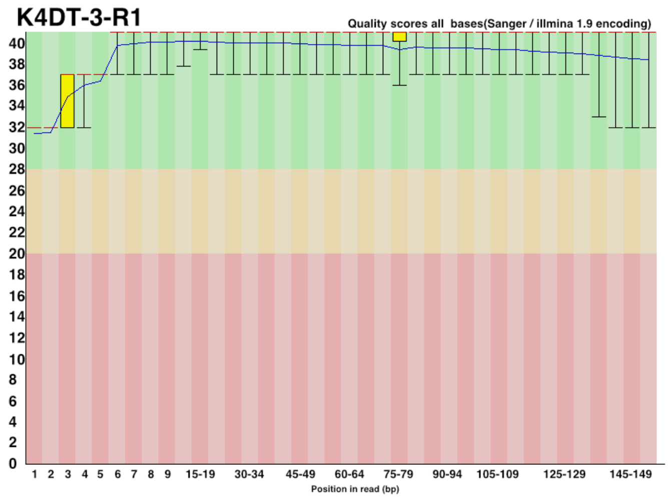
K4DT-3-R1
Quality scores all bases(Sanger / illmina 1.9 encoding)
40 38 36 34 32 30 28 26 24 22 20 18 16 14 12 10 8 6 4 2 0
1 2 3 4 5 6 7 8 9 15-19 30-34 45-49 60-64 75-79 90-94 105-109 125-129 145-149
Position in read (bp)

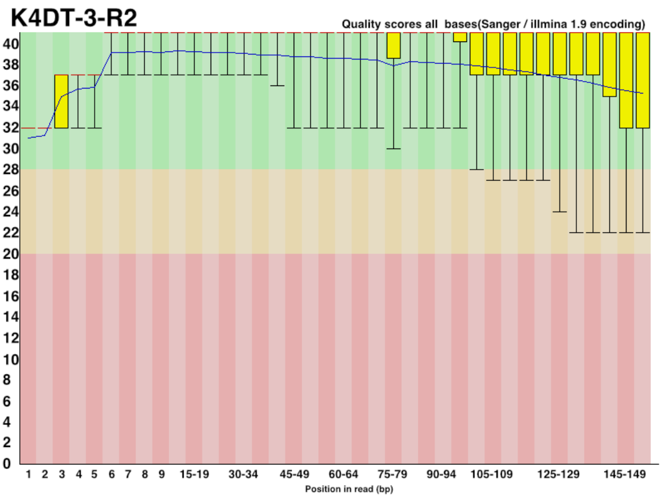
K4DT-3-R2
Quality scores all bases(Sanger / illmina 1.9 encoding)
40 38 36 34 32 30 28 26 24 22 20 18 16 14 12 10 8 6 4 2 0
1 2 3 4 5 6 7 8 9 15-19 30-34 45-49 60-64 75-79 90-94 105-109 125-129 145-149
Position in read (bp)

SV40-1-R1 no serum

Quality scores all bases(Sanger / illmina 1.9 encoding)

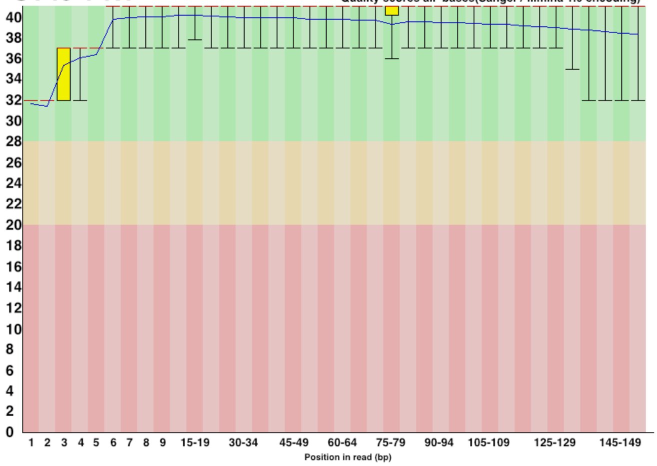

SV40-1-R2 no serum

Quality scores all bases(Sanger / illmina 1.9 encoding)

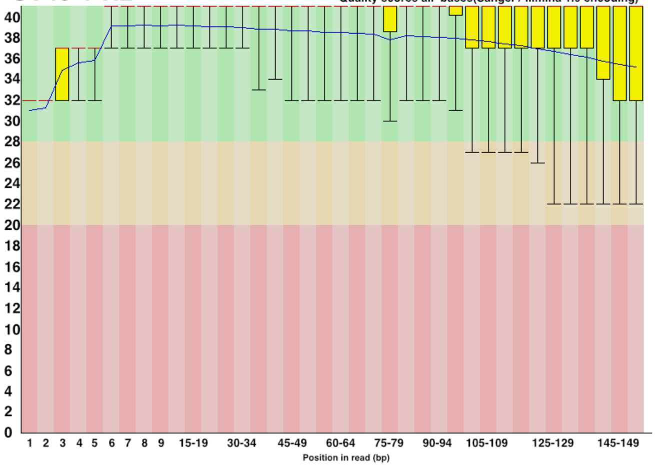

SV40-2-R1 no serum

Quality scores all bases(Sanger / illmina 1.9 encoding)

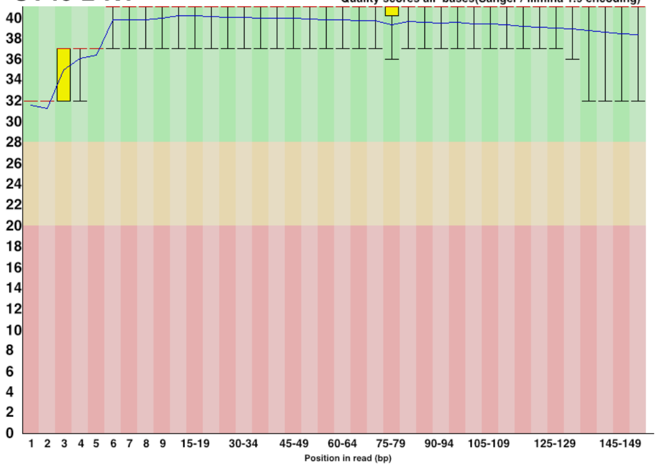

SV40-2-R2 no serum

Quality scores all bases(Sanger / illmina 1.9 encoding)

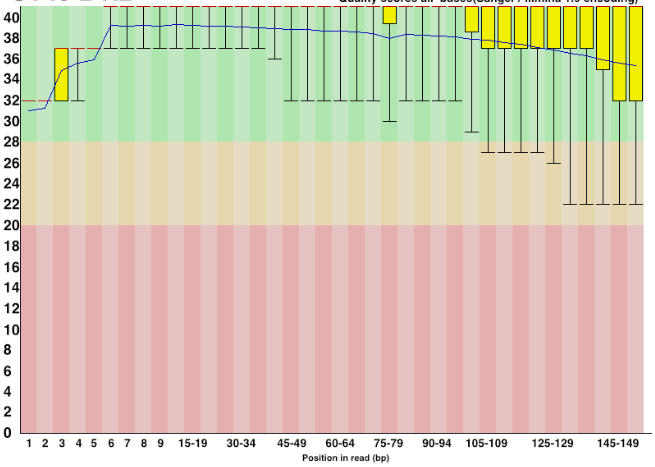

SV40-3-R1 no serum

Quality scores all bases(Sanger / illmina 1.9 encoding)

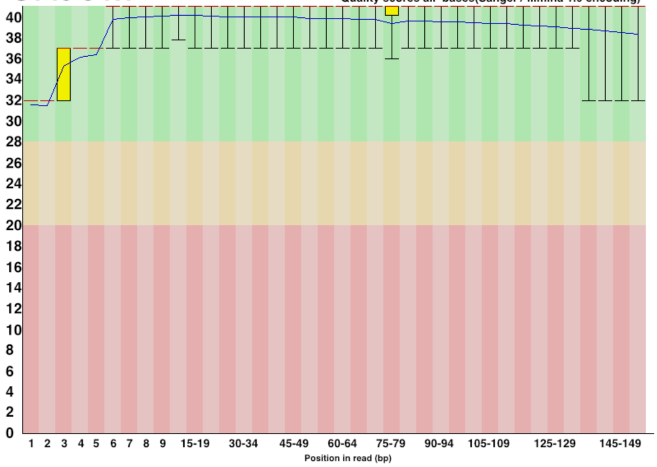

SV40-3-R2 no serum

Quality scores all bases(Sanger / illmina 1.9 encoding)

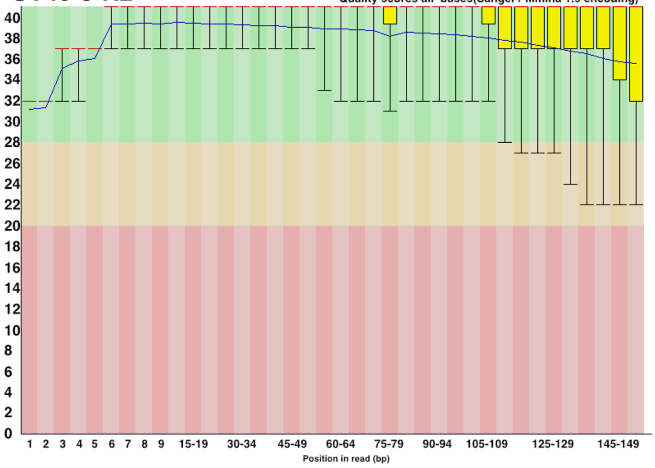

SV40-4-R1 serum

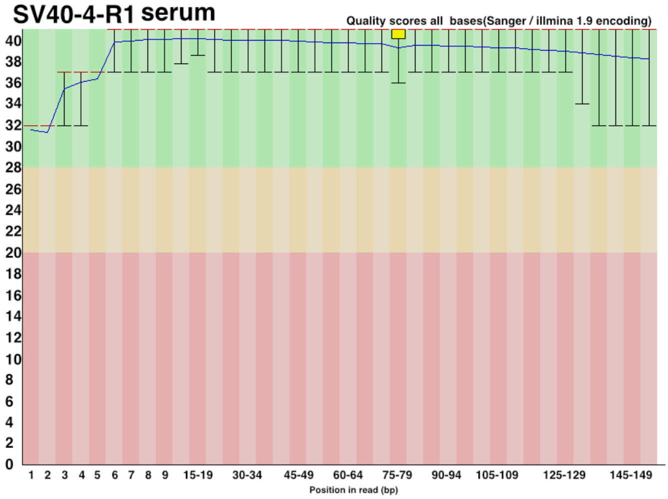

SV40-4-R2 serum

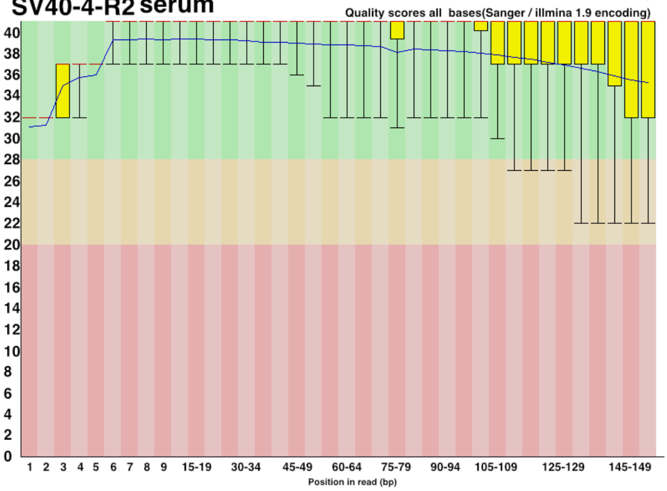

SV40-5-R1 serum

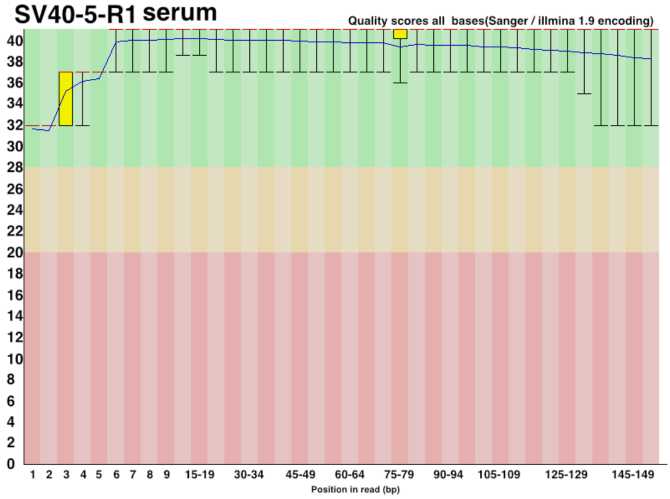

SV40-5-R2 serum

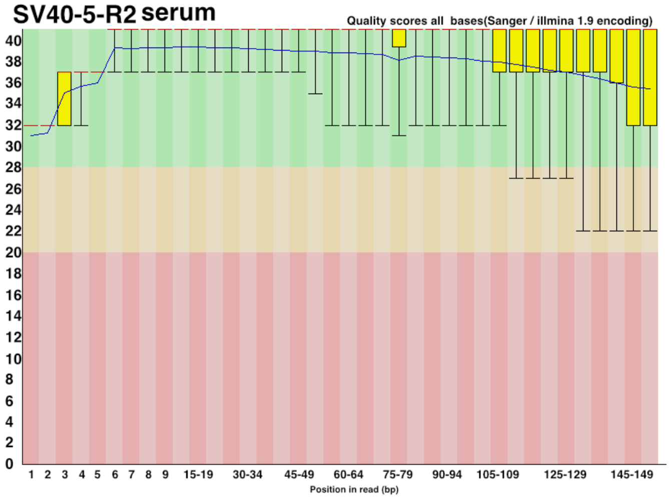

SV40-6-R1 serum

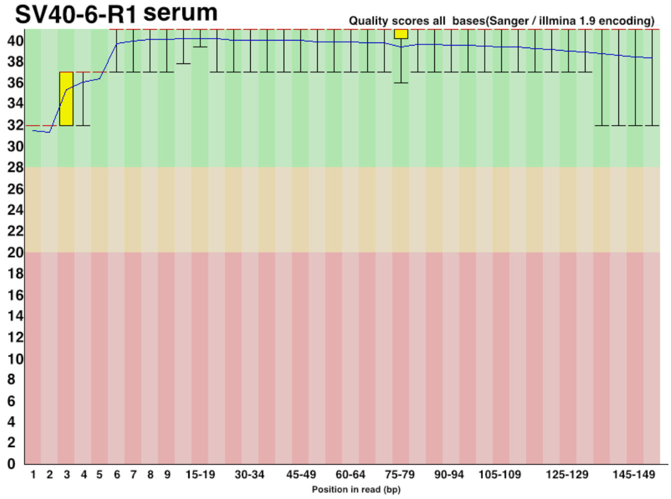

SV40-6-R2 serum

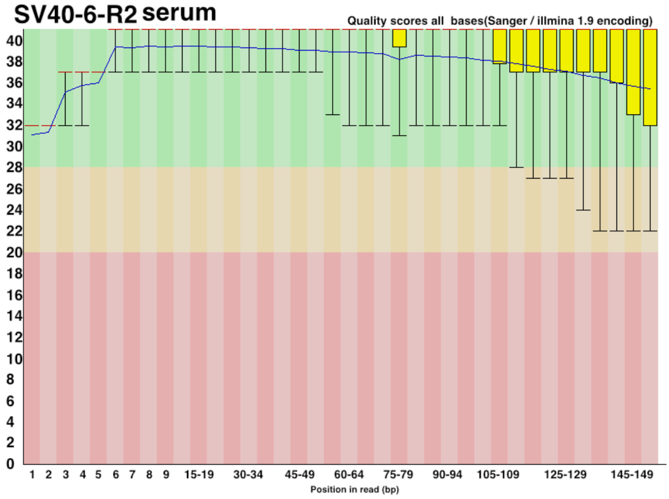

Cell cycle

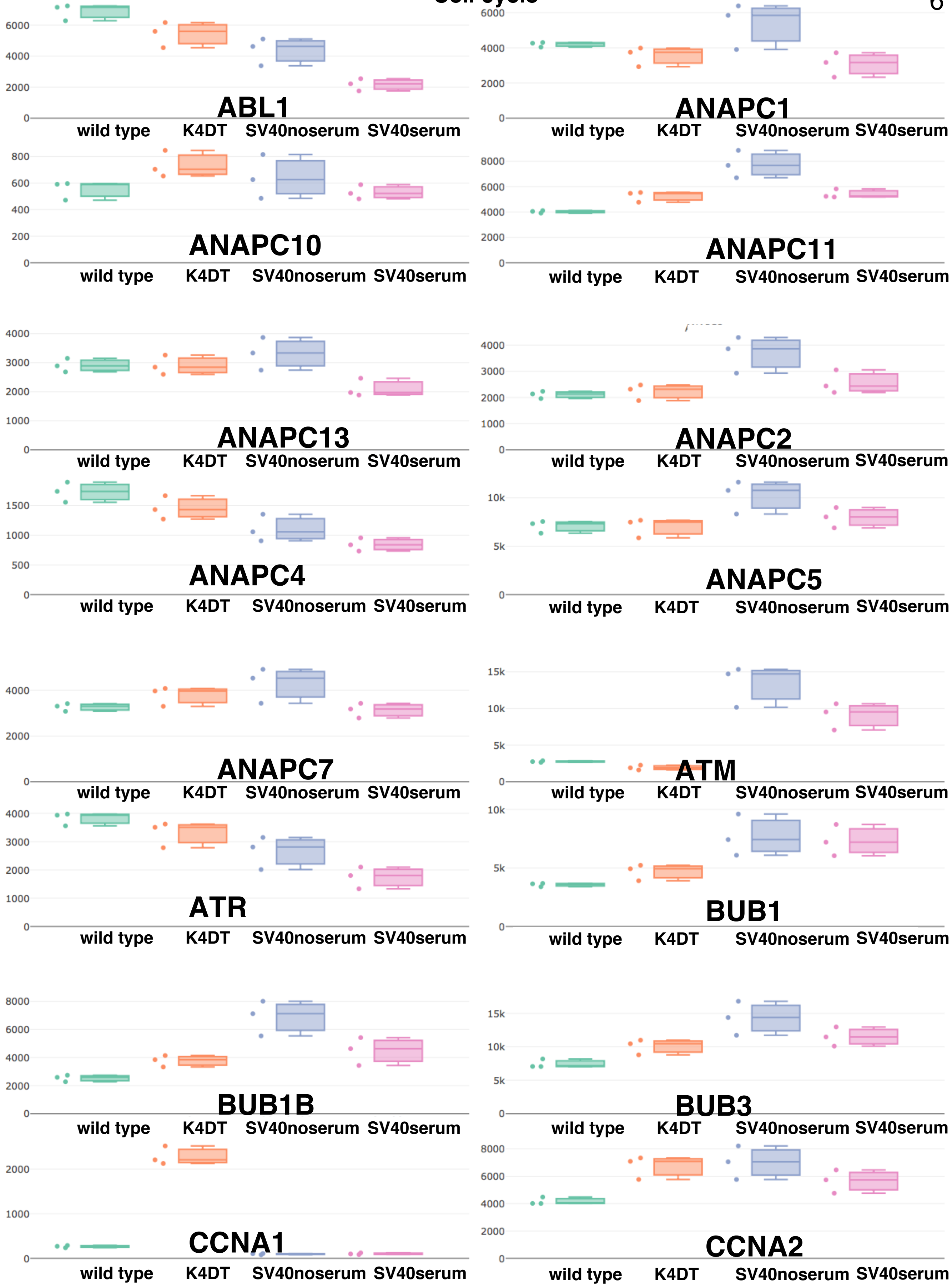
ABL1
ANAPC1
ANAPC10
ANAPC11
ANAPC13
ANAPC2
ANAPC4
ANAPC5
ANAPC7
ATM
ATR
BUB1
BUB1B
BUB3
CCNA1
CCNA2
wild type
K4DT
SV40noserum
SV40serum

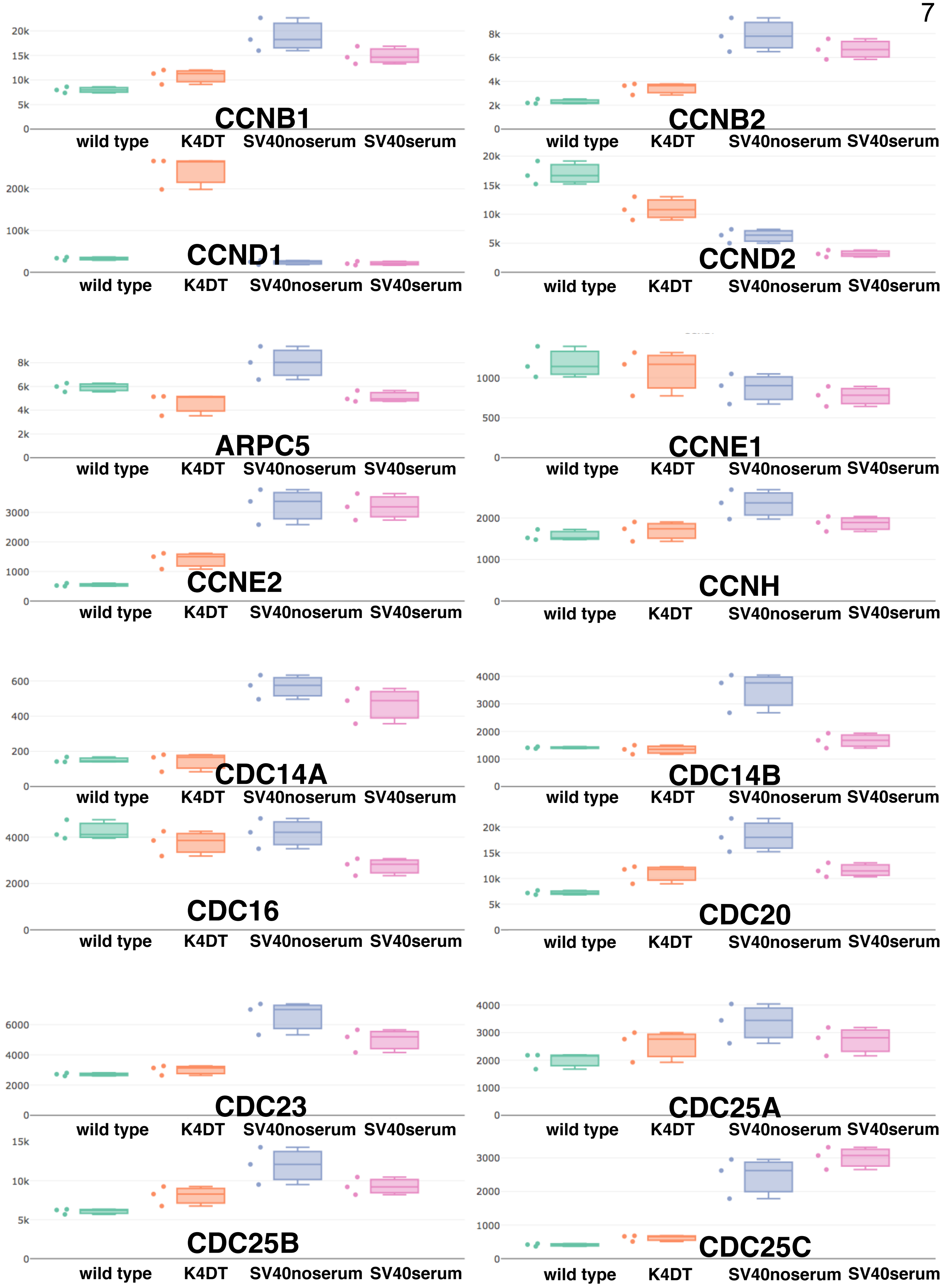
CCNB1
20k
15k
10k
5k
0
wild type
K4DT
SV40noserum
SV40serum
CCNB2
8k
6k
4k
2k
0
wild type
K4DT
SV40noserum
SV40serum
CCND1
200k
100k
0
wild type
K4DT
SV40noserum
SV40serum
CCND2
20k
15k
10k
5k
0
wild type
K4DT
SV40noserum
SV40serum
ARPC5
8k
6k
4k
2k
0
wild type
K4DT
SV40noserum
SV40serum
CCNE1
1000
500
0
wild type
K4DT
SV40noserum
SV40serum
CCNE2
3000
2000
1000
0
wild type
K4DT
SV40noserum
SV40serum
CCNH
2000
1000
0
wild type
K4DT
SV40noserum
SV40serum
CDC14A
600
400
200
0
wild type
K4DT
SV40noserum
SV40serum
CDC14B
4000
3000
2000
1000
0
wild type
K4DT
SV40noserum
SV40serum
CDC16
4000
2000
0
wild type
K4DT
SV40noserum
SV40serum
CDC20
20k
15k
10k
5k
0
wild type
K4DT
SV40noserum
SV40serum
CDC23
6000
4000
2000
0
wild type
K4DT
SV40noserum
SV40serum
CDC25A
4000
3000
2000
1000
0
wild type
K4DT
SV40noserum
SV40serum
CDC25B
15k
10k
5k
0
wild type
K4DT
SV40noserum
SV40serum
CDC25C
3000
2000
1000
0
wild type
K4DT
SV40noserum
SV40serum

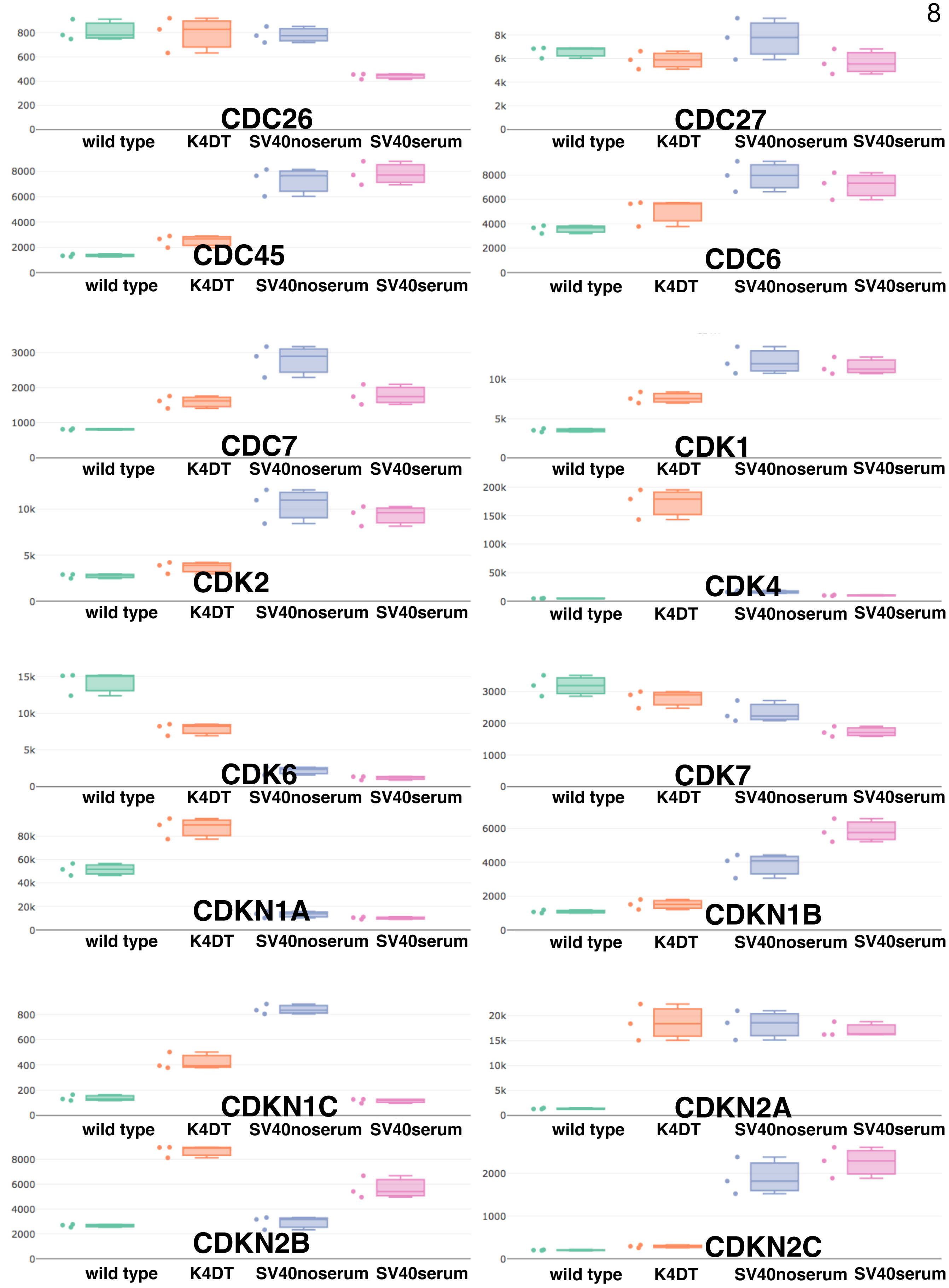
CDC26
0
200
400
600
800
wild type
K4DT
SV40noserum
SV40serum
CDC27
0
2k
4k
6k
8k
CDC45
0
2000
4000
6000
8000
CDC6
CDC7
0
1000
2000
3000
CDK1
5k
10k
CDK2
CDK4
50k
100k
150k
200k
CDK6
15k
CDK7
CDKN1A
20k
40k
60k
80k
CDKN1B
CDKN1C
CDKN2A
15k
20k
CDKN2B
CDKN2C

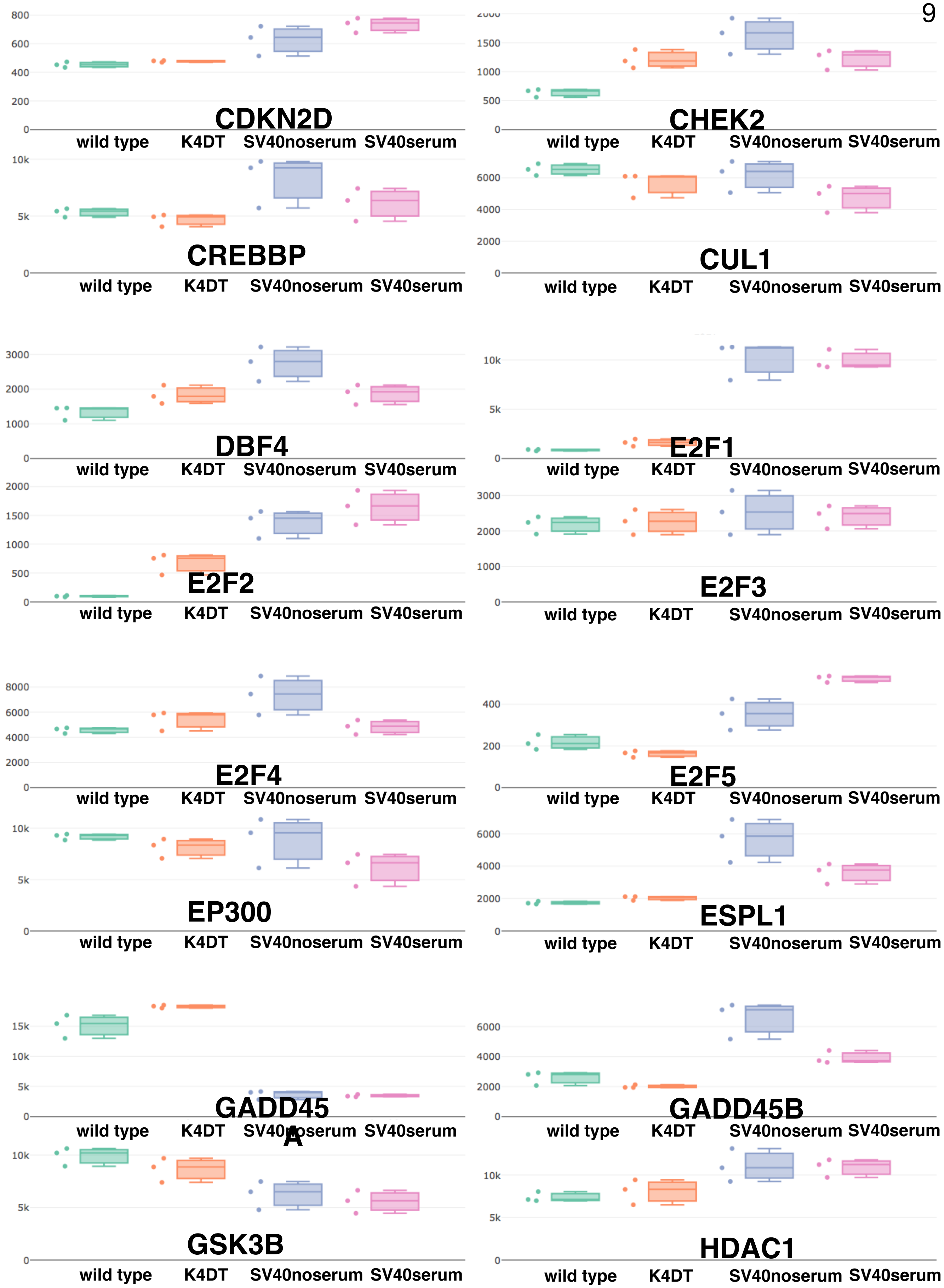
CDKN2D
0
200
400
600
800
wild type
K4DT
SV40noserum
SV40serum
CHEK2
0
500
1000
1500
wild type
K4DT
SV40noserum
SV40serum
CREBBP
0
5k
10k
wild type
K4DT
SV40noserum
SV40serum
CUL1
0
2000
4000
6000
wild type
K4DT
SV40noserum
SV40serum
DBF4
0
1000
2000
3000
wild type
K4DT
SV40noserum
SV40serum
E2F1
0
5k
10k
wild type
K4DT
SV40noserum
SV40serum
E2F2
0
500
1000
1500
2000
wild type
K4DT
SV40noserum
SV40serum
E2F3
0
1000
2000
3000
wild type
K4DT
SV40noserum
SV40serum
E2F4
0
2000
4000
6000
8000
wild type
K4DT
SV40noserum
SV40serum
E2F5
0
200
400
wild type
K4DT
SV40noserum
SV40serum
EP300
0
5k
10k
wild type
K4DT
SV40noserum
SV40serum
ESPL1
0
2000
4000
6000
wild type
K4DT
SV40noserum
SV40serum
GADD45
A
0
5k
10k
15k
wild type
K4DT
SV40noserum
SV40serum
GADD45B
0
2000
4000
6000
wild type
K4DT
SV40noserum
SV40serum
GSK3B
0
5k
10k
wild type
K4DT
SV40noserum
SV40serum
HDAC1
0
5k
10k
wild type
K4DT
SV40noserum
SV40serum

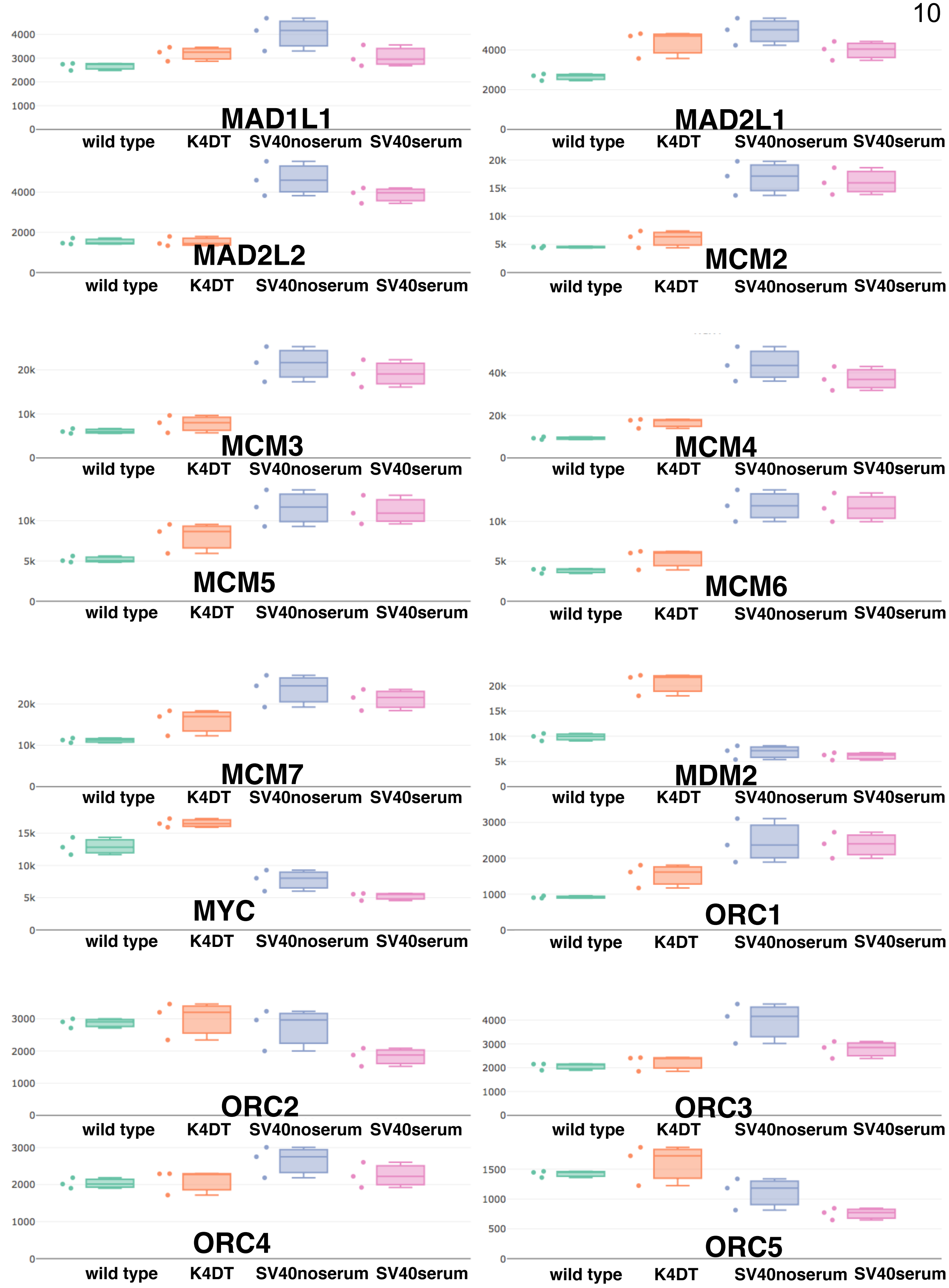
MAD1L1
0
1000
2000
3000
4000
wild type
K4DT
SV40noserum
SV40serum
MAD2L1
0
2000
4000
wild type
K4DT
SV40noserum
SV40serum
MAD2L2
0
2000
4000
wild type
K4DT
SV40noserum
SV40serum
MCM2
0
5k
10k
15k
20k
wild type
K4DT
SV40noserum
SV40serum
MCM3
0
10k
20k
wild type
K4DT
SV40noserum
SV40serum
MCM4
0
20k
40k
wild type
K4DT
SV40noserum
SV40serum
MCM5
0
5k
10k
wild type
K4DT
SV40noserum
SV40serum
MCM6
0
5k
10k
wild type
K4DT
SV40noserum
SV40serum
MCM7
0
10k
20k
wild type
K4DT
SV40noserum
SV40serum
MDM2
0
5k
10k
15k
20k
wild type
K4DT
SV40noserum
SV40serum
MYC
0
5k
10k
15k
wild type
K4DT
SV40noserum
SV40serum
ORC1
0
1000
2000
3000
wild type
K4DT
SV40noserum
SV40serum
ORC2
0
1000
2000
3000
wild type
K4DT
SV40noserum
SV40serum
ORC3
0
1000
2000
3000
4000
wild type
K4DT
SV40noserum
SV40serum
ORC4
0
1000
2000
3000
wild type
K4DT
SV40noserum
SV40serum
ORC5
0
500
1000
1500
wild type
K4DT
SV40noserum
SV40serum

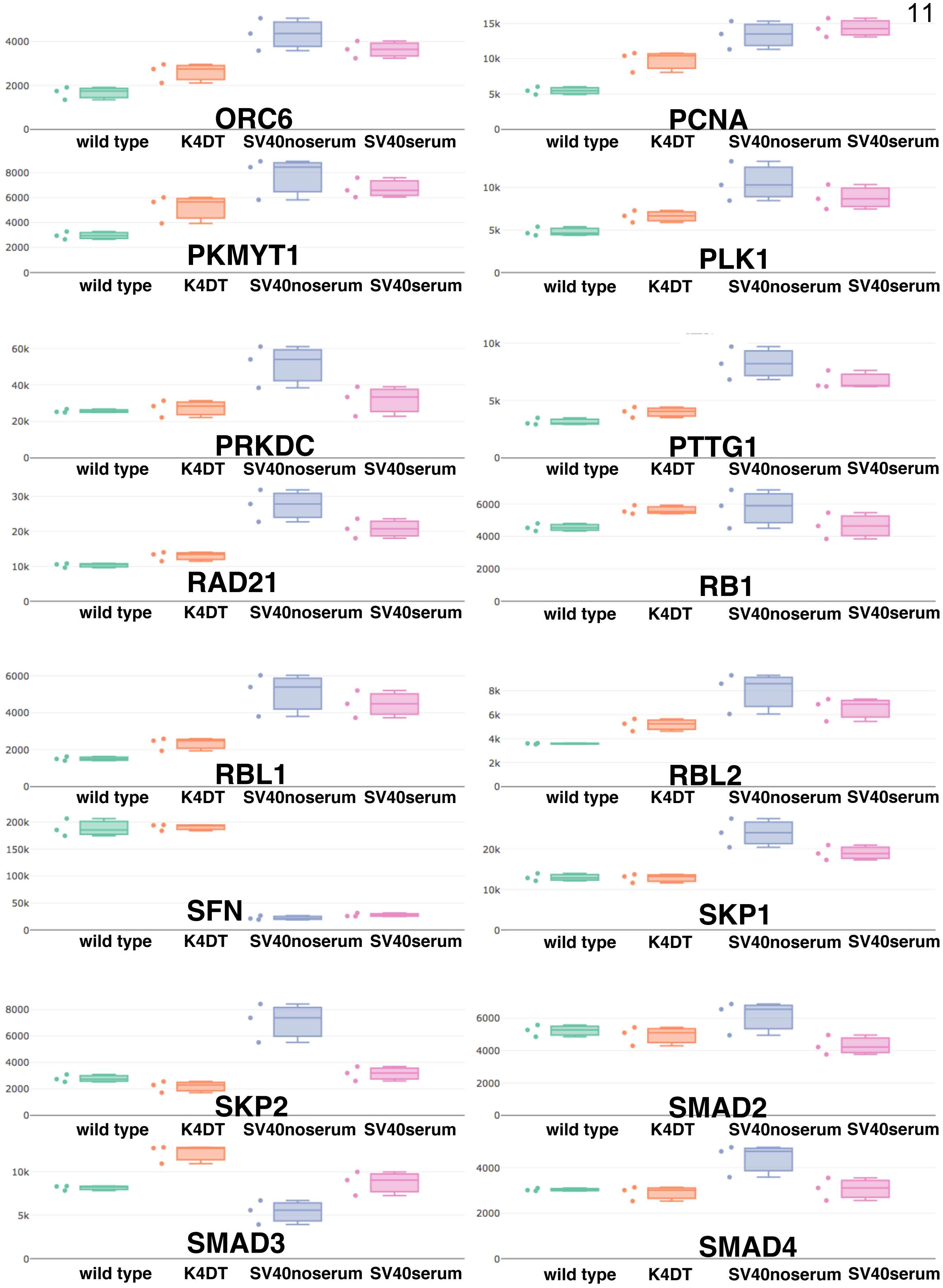
ORC6
PCNA
PKMYT1
PLK1
PRKDC
PTTG1
RAD21
RB1
RBL1
RBL2
SFN
SKP1
SKP2
SMAD2
SMAD3
SMAD4
wild type
K4DT
SV40noserum
SV40serum

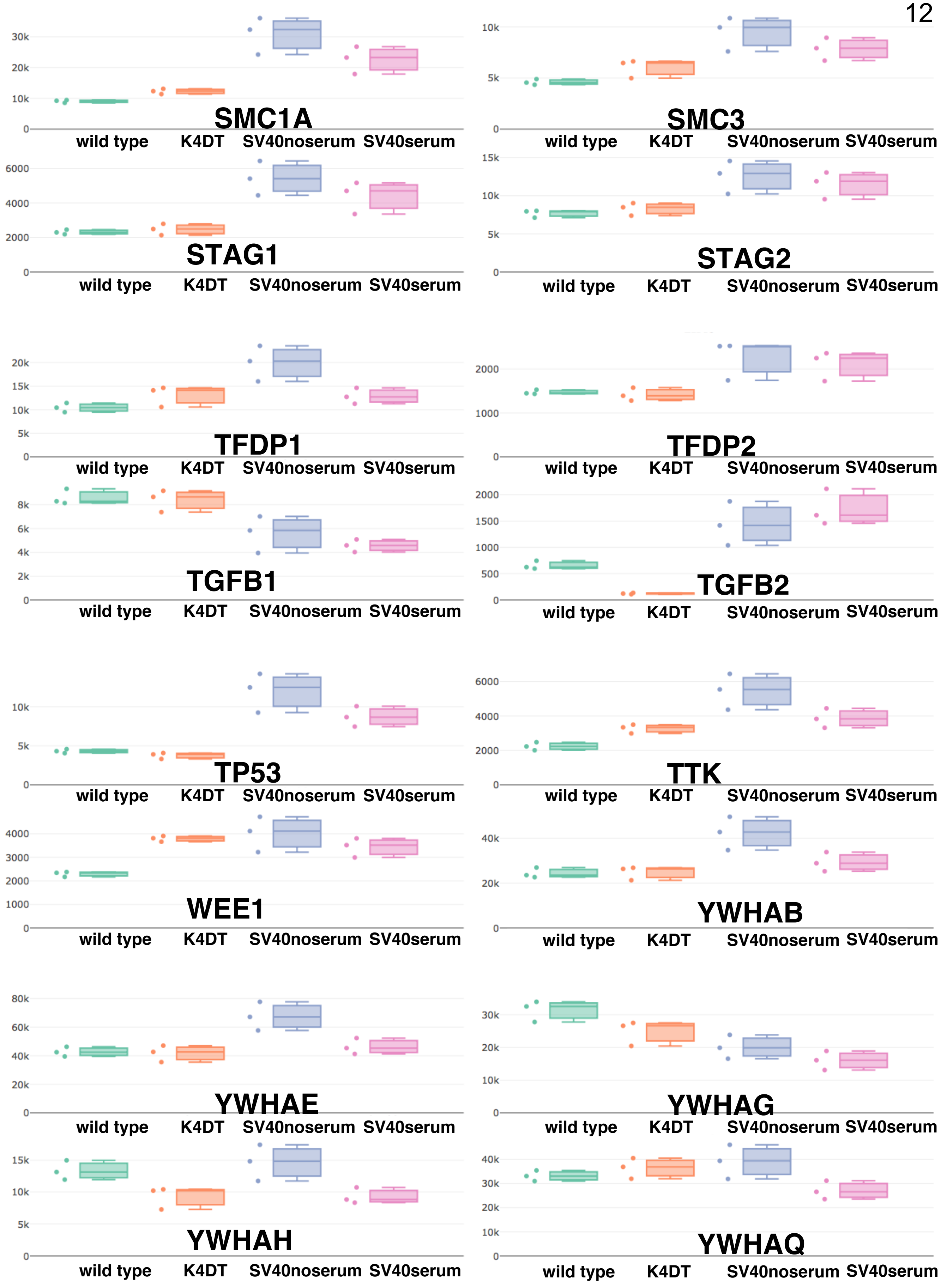
SMC1A
0 10k 20k 30k
wild type K4DT SV40noserum SV40serum
SMC3
0 5k 10k
wild type K4DT SV40noserum SV40serum
STAG1
0 2000 4000 6000
wild type K4DT SV40noserum SV40serum
STAG2
0 5k 10k 15k
wild type K4DT SV40noserum SV40serum
TFDP1
0 5k 10k 15k 20k
wild type K4DT SV40noserum SV40serum
TFDP2
0 1000 2000
wild type K4DT SV40noserum SV40serum
TGFB1
0 2k 4k 6k 8k
wild type K4DT SV40noserum SV40serum
TGFB2
0 500 1000 1500 2000
wild type K4DT SV40noserum SV40serum
TP53
0 5k 10k
wild type K4DT SV40noserum SV40serum
TTK
0 2000 4000 6000
wild type K4DT SV40noserum SV40serum
WEE1
0 1000 2000 3000 4000
wild type K4DT SV40noserum SV40serum
YWHAB
0 20k 40k
wild type K4DT SV40noserum SV40serum
YWHAE
0 20k 40k 60k 80k
wild type K4DT SV40noserum SV40serum
YWHAG
0 10k 20k 30k
wild type K4DT SV40noserum SV40serum
YWHAH
0 5k 10k 15k
wild type K4DT SV40noserum SV40serum
YWHAQ
0 10k 20k 30k 40k
wild type K4DT SV40noserum SV40serum

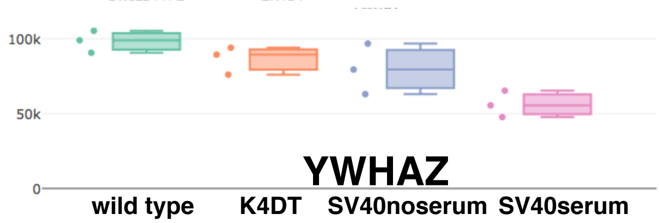
100k
50k
0
YWHAZ
wild type
K4DT
SV40noserum
SV40serum

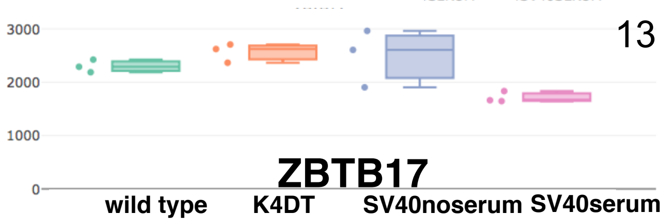
3000
2000
1000
0
ZBTB17
wild type
K4DT
SV40noserum
SV40serum

Ubiquitin mediated proteolysis

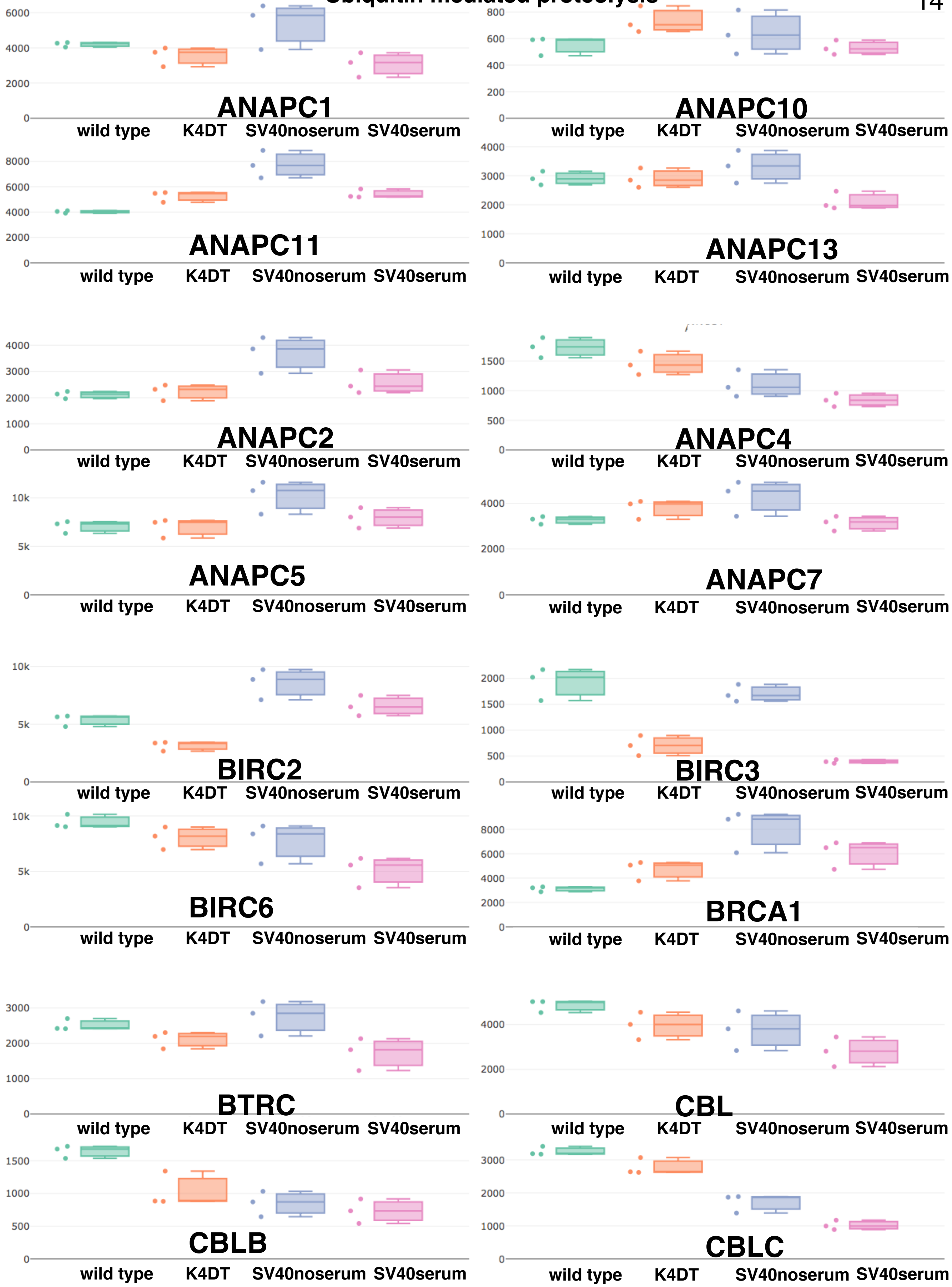

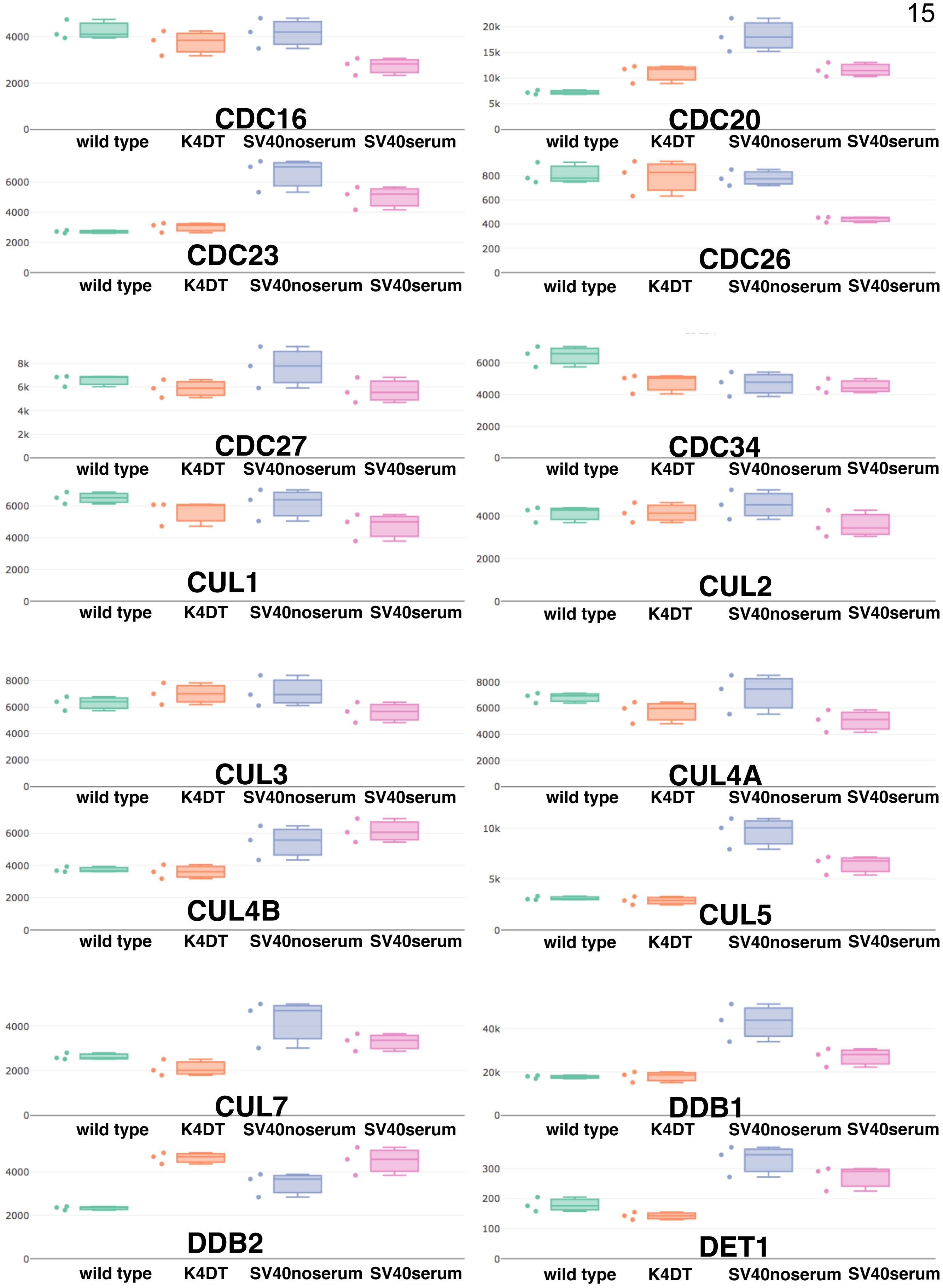
4000
2000
0
CDC16
wild type
K4DT
SV40noserum
SV40serum
20k
15k
10k
5k
0
CDC20
wild type
K4DT
SV40noserum
SV40serum
6000
4000
2000
0
CDC23
wild type
K4DT
SV40noserum
SV40serum
800
600
400
200
0
CDC26
wild type
K4DT
SV40noserum
SV40serum
8k
6k
4k
2k
0
CDC27
wild type
K4DT
SV40noserum
SV40serum
6000
4000
2000
0
CDC34
wild type
K4DT
SV40noserum
SV40serum
6000
4000
2000
0
CUL1
wild type
K4DT
SV40noserum
SV40serum
4000
2000
0
CUL2
wild type
K4DT
SV40noserum
SV40serum
8000
6000
4000
2000
0
CUL3
wild type
K4DT
SV40noserum
SV40serum
8000
6000
4000
2000
0
CUL4A
wild type
K4DT
SV40noserum
SV40serum
6000
4000
2000
0
CUL4B
wild type
K4DT
SV40noserum
SV40serum
10k
5k
0
CUL5
wild type
K4DT
SV40noserum
SV40serum
4000
2000
0
CUL7
wild type
K4DT
SV40noserum
SV40serum
40k
20k
0
DDB1
wild type
K4DT
SV40noserum
SV40serum
4000
2000
0
DDB2
wild type
K4DT
SV40noserum
SV40serum
300
200
100
0
DET1
wild type
K4DT
SV40noserum
SV40serum

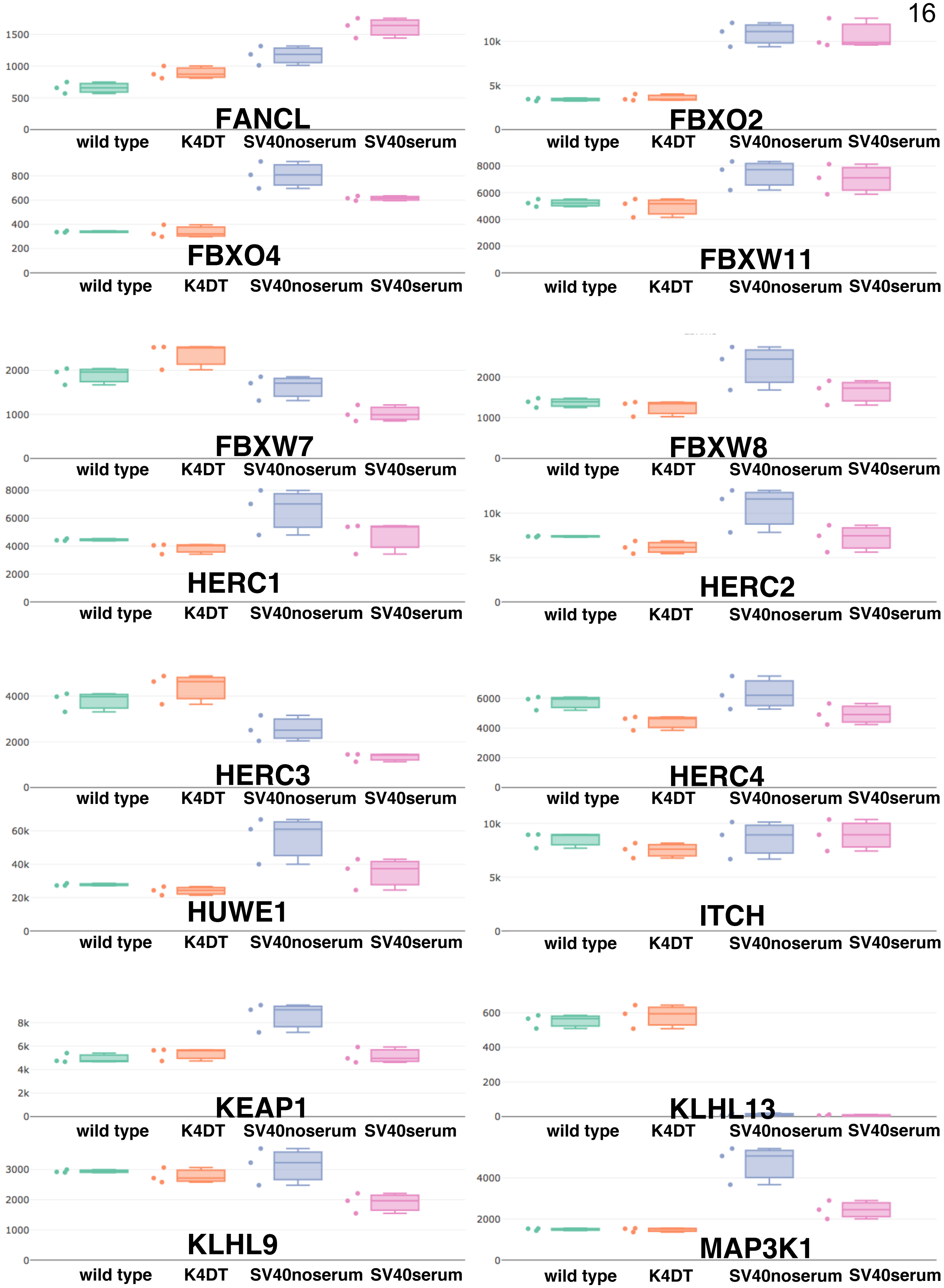
FANCL
0
500
1000
1500
wild type
K4DT
SV40noserum
SV40serum
FBXO2
0
5k
10k
wild type
K4DT
SV40noserum
SV40serum
FBXO4
0
200
400
600
800
wild type
K4DT
SV40noserum
SV40serum
FBXW11
0
2000
4000
6000
8000
wild type
K4DT
SV40noserum
SV40serum
FBXW7
0
1000
2000
wild type
K4DT
SV40noserum
SV40serum
FBXW8
0
1000
2000
wild type
K4DT
SV40noserum
SV40serum
HERC1
0
2000
4000
6000
8000
wild type
K4DT
SV40noserum
SV40serum
HERC2
0
5k
10k
wild type
K4DT
SV40noserum
SV40serum
HERC3
0
2000
4000
wild type
K4DT
SV40noserum
SV40serum
HERC4
0
2000
4000
6000
wild type
K4DT
SV40noserum
SV40serum
HUWE1
0
20k
40k
60k
wild type
K4DT
SV40noserum
SV40serum
ITCH
0
5k
10k
wild type
K4DT
SV40noserum
SV40serum
KEAP1
0
2k
4k
6k
8k
wild type
K4DT
SV40noserum
SV40serum
KLHL13
0
200
400
600
wild type
K4DT
SV40noserum
SV40serum
KLHL9
0
1000
2000
3000
wild type
K4DT
SV40noserum
SV40serum
MAP3K1
0
2000
4000
wild type
K4DT
SV40noserum
SV40serum

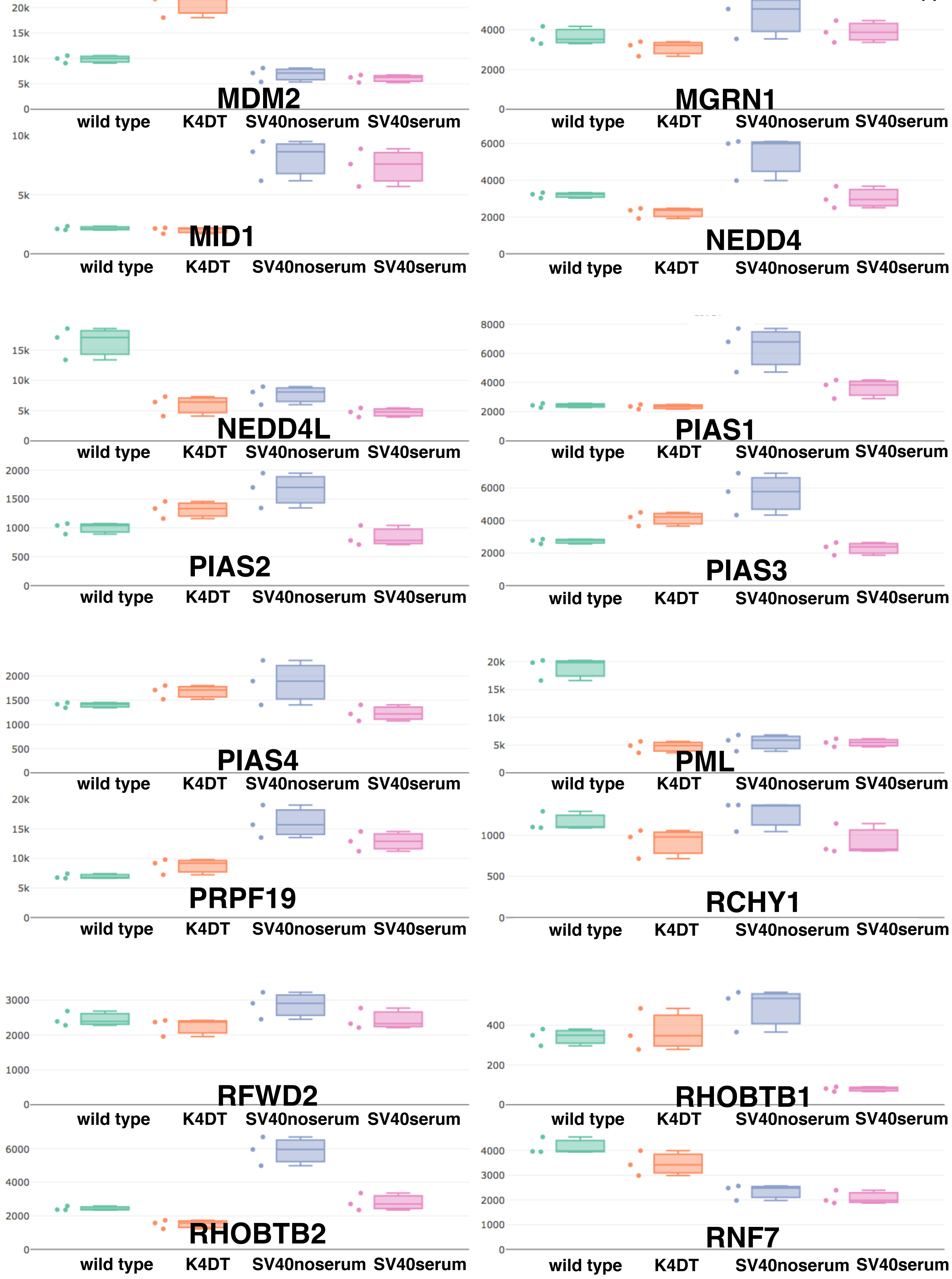

MDM2
wild type K4DT SV40noserum SV40serum
MGRN1
wild type K4DT SV40noserum SV40serum
MID1
wild type K4DT SV40noserum SV40serum
NEDD4
wild type K4DT SV40noserum SV40serum
NEDD4L
wild type K4DT SV40noserum SV40serum
PIAS1
wild type K4DT SV40noserum SV40serum
PIAS2
wild type K4DT SV40noserum SV40serum
PIAS3
wild type K4DT SV40noserum SV40serum
PIAS4
wild type K4DT SV40noserum SV40serum
PML
wild type K4DT SV40noserum SV40serum
PRPF19
wild type K4DT SV40noserum SV40serum
RCHY1
wild type K4DT SV40noserum SV40serum
RFWD2
wild type K4DT SV40noserum SV40serum
RHOBTB1
wild type K4DT SV40noserum SV40serum
RHOBTB2
wild type K4DT SV40noserum SV40serum
RNF7
wild type K4DT SV40noserum SV40serum

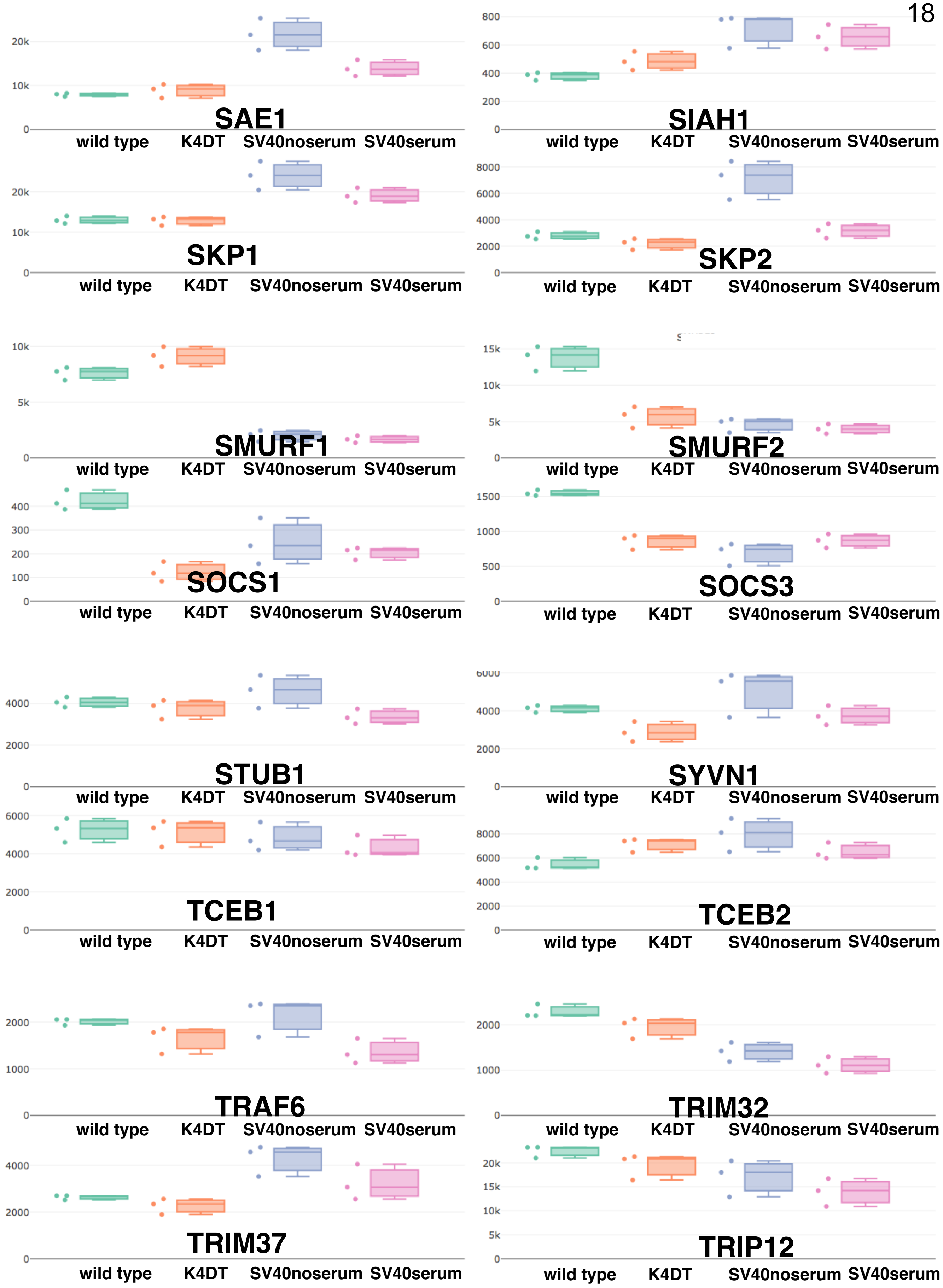
SAE1
20k
10k
0
wild type
K4DT
SV40noserum
SV40serum
SIAH1
800
600
400
200
0
wild type
K4DT
SV40noserum
SV40serum
SKP1
20k
10k
0
wild type
K4DT
SV40noserum
SV40serum
SKP2
8000
6000
4000
2000
0
wild type
K4DT
SV40noserum
SV40serum
SMURF1
10k
5k
0
wild type
K4DT
SV40noserum
SV40serum
SMURF2
15k
10k
5k
0
wild type
K4DT
SV40noserum
SV40serum
SOCS1
400
300
200
100
0
wild type
K4DT
SV40noserum
SV40serum
SOCS3
1500
1000
500
0
wild type
K4DT
SV40noserum
SV40serum
STUB1
4000
2000
0
wild type
K4DT
SV40noserum
SV40serum
SYVN1
6000
4000
2000
0
wild type
K4DT
SV40noserum
SV40serum
TCEB1
6000
4000
2000
0
wild type
K4DT
SV40noserum
SV40serum
TCEB2
8000
6000
4000
2000
0
wild type
K4DT
SV40noserum
SV40serum
TRAF6
2000
1000
0
wild type
K4DT
SV40noserum
SV40serum
TRIM32
2000
1000
0
wild type
K4DT
SV40noserum
SV40serum
TRIM37
4000
2000
0
wild type
K4DT
SV40noserum
SV40serum
TRIP12
20k
15k
10k
5k
0
wild type
K4DT
SV40noserum
SV40serum

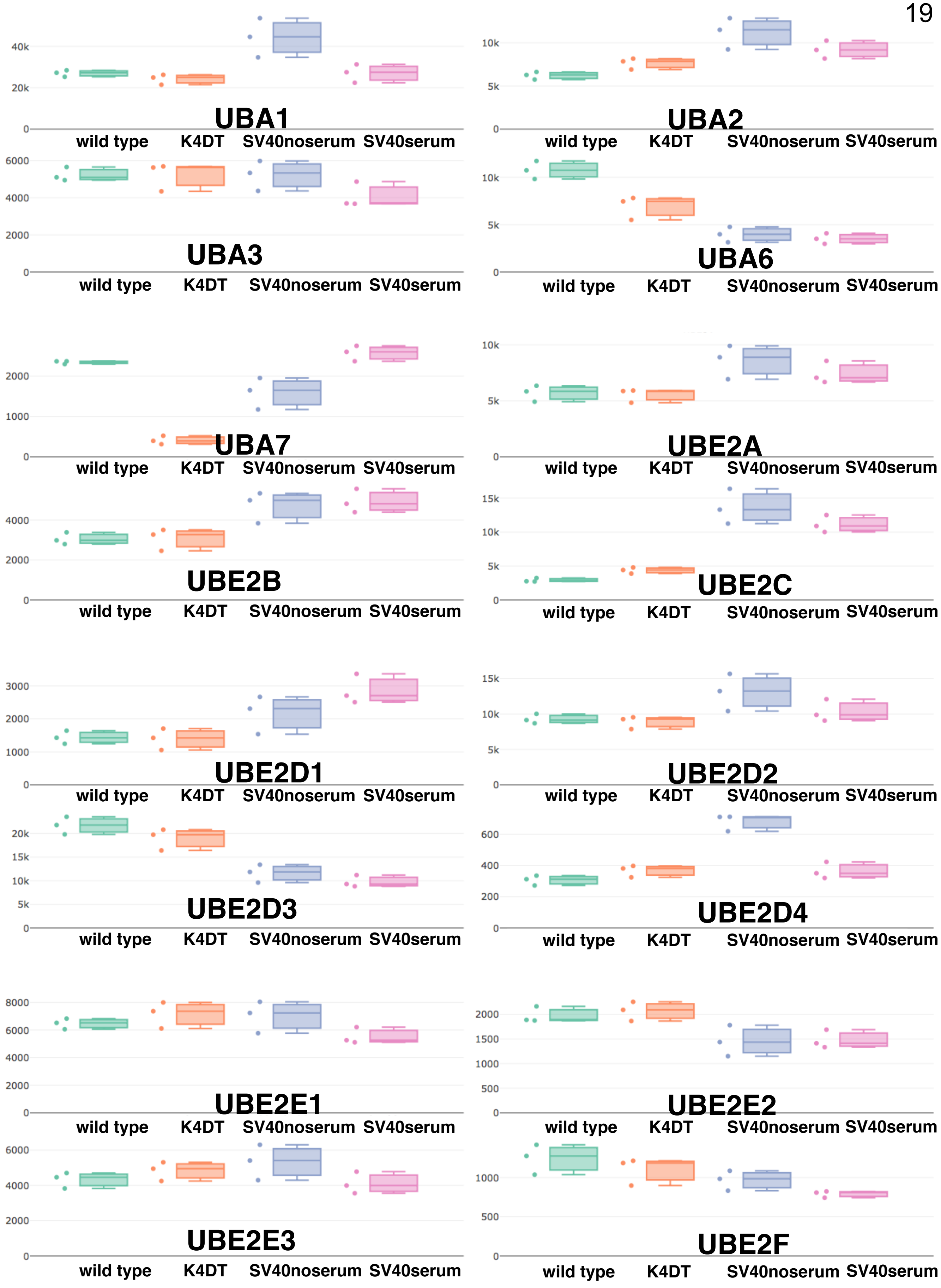
UBA1
UBA2
UBA3
UBA6
UBA7
UBE2A
UBE2B
UBE2C
UBE2D1
UBE2D2
UBE2D3
UBE2D4
UBE2E1
UBE2E2
UBE2E3
UBE2F
wild type
K4DT
SV40noserum
SV40serum

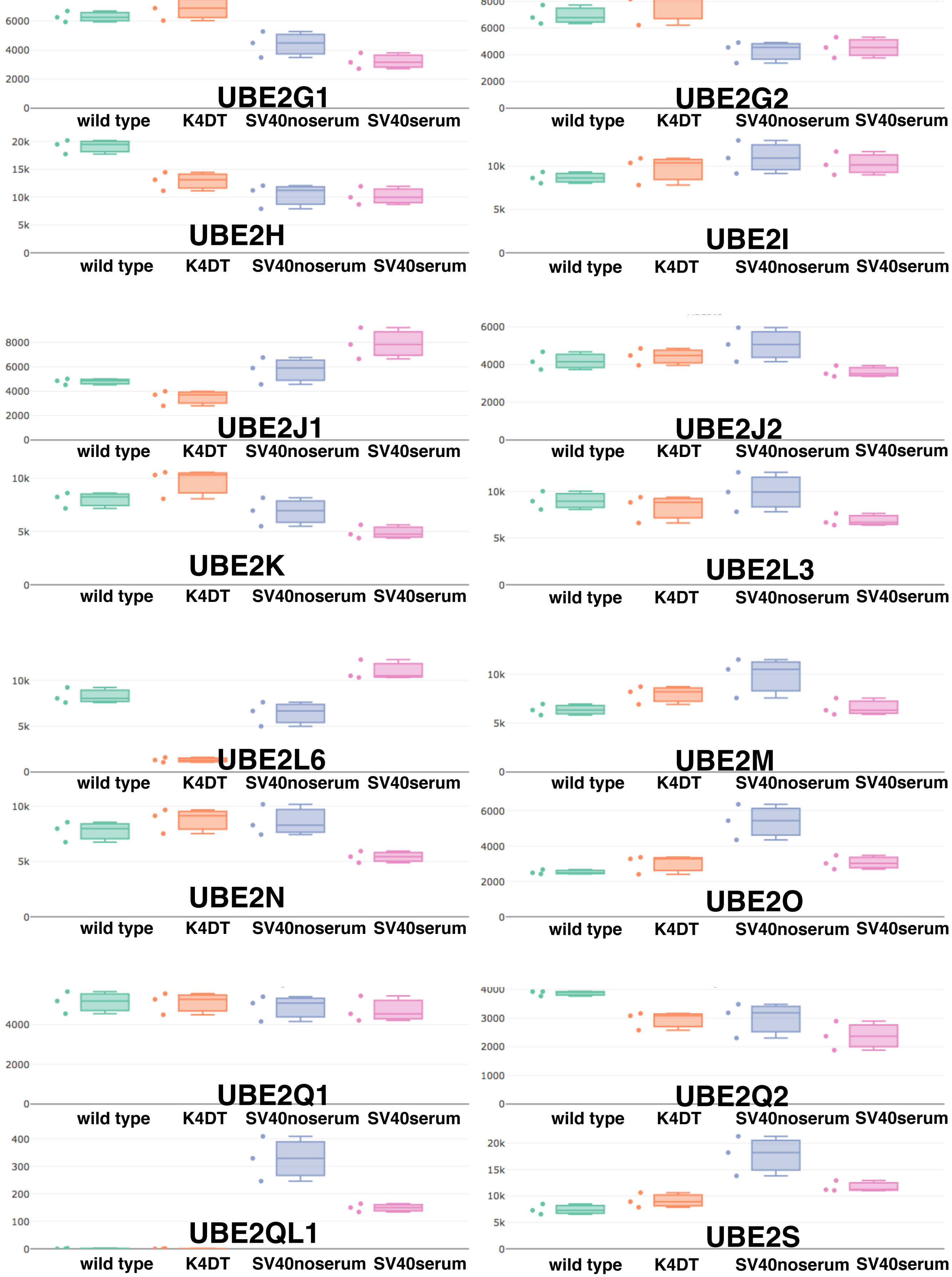
UBE2G1
wild type
K4DT
SV40noserum
SV40serum
UBE2G2
UBE2H
UBE2I
UBE2J1
UBE2J2
UBE2K
UBE2L3
UBE2L6
UBE2M
UBE2N
UBE2O
UBE2Q1
UBE2Q2
UBE2QL1
UBE2S

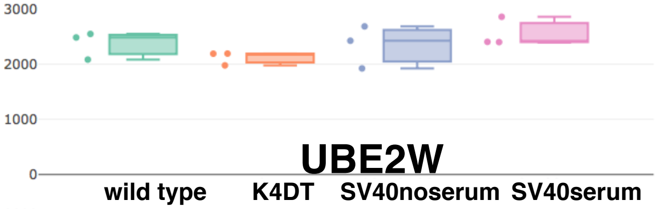
3000
2000
1000
0
UBE2W
wild type
K4DT
SV40noserum
SV40serum

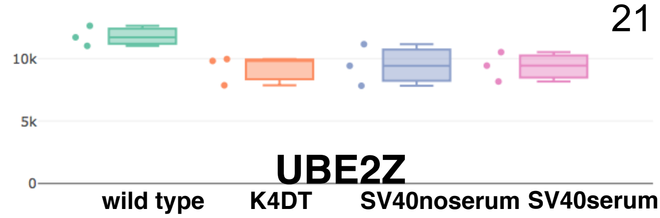
10k
5k
0
UBE2Z
wild type
K4DT
SV40noserum
SV40serum

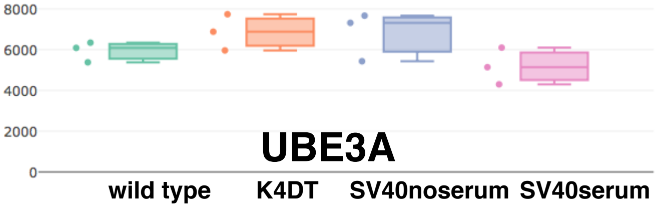
8000
6000
4000
2000
0
UBE3A
wild type
K4DT
SV40noserum
SV40serum

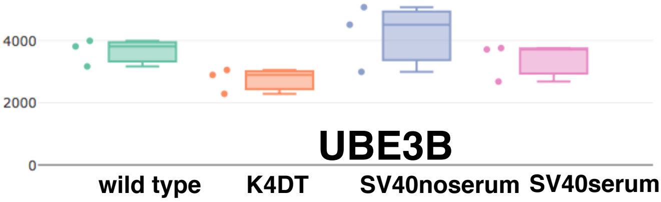
4000
2000
0
UBE3B
wild type
K4DT
SV40noserum
SV40serum

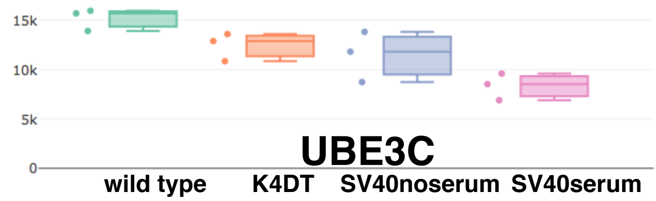
15k
10k
5k
0
UBE3C
wild type
K4DT
SV40noserum
SV40serum

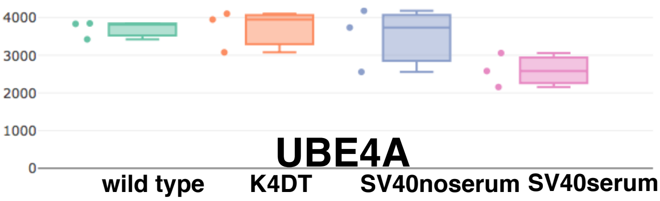
4000
3000
2000
1000
0
UBE4A
wild type
K4DT
SV40noserum
SV40serum

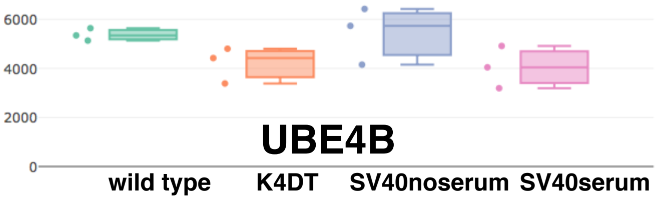
6000
4000
2000
0
UBE4B
wild type
K4DT
SV40noserum
SV40serum

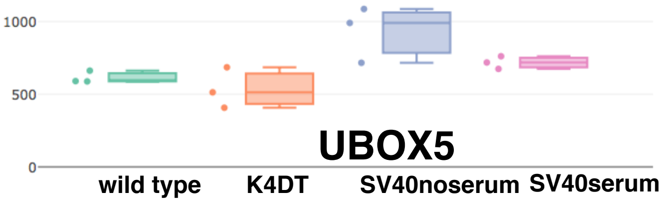
1000
500
0
UBOX5
wild type
K4DT
SV40noserum
SV40serum

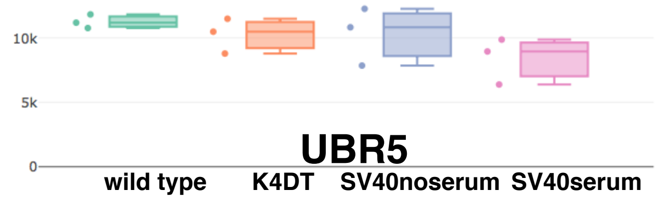
10k
5k
0
UBR5
wild type
K4DT
SV40noserum
SV40serum

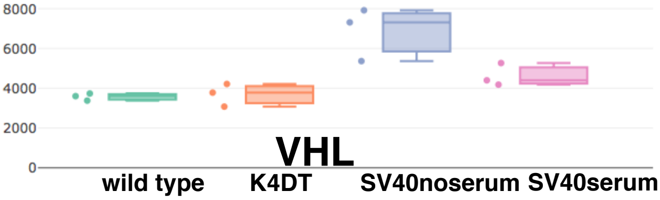
8000
6000
4000
2000
0
VHL
wild type
K4DT
SV40noserum
SV40serum

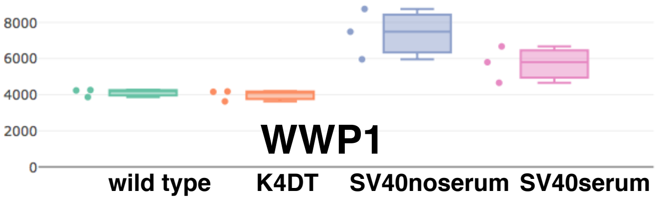
8000
6000
4000
2000
0
WWP1
wild type
K4DT
SV40noserum
SV40serum

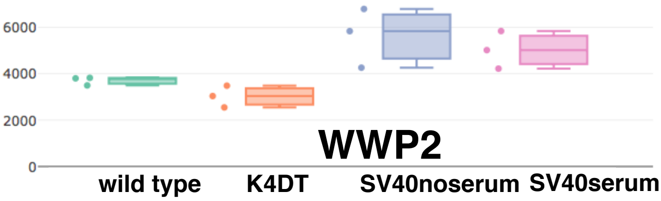
6000
4000
2000
0
WWP2
wild type
K4DT
SV40noserum
SV40serum

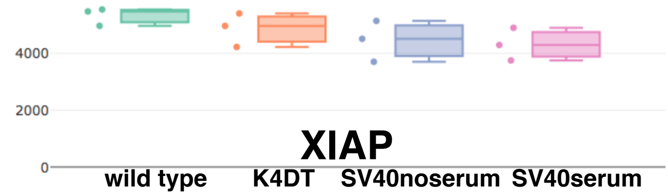
4000
2000
0
XIAP
wild type
K4DT
SV40noserum
SV40serum

a

Chromosome analysis of SV40T expressing or K4DT corneal epithelial cells

Cell line	number of chromosome per cell		
	45	46	62
K4DT		49	1

Cell line	number of chromosome per cell					
	59	60	61	62	63	64
SV40	2	3	9	8	24	4

b

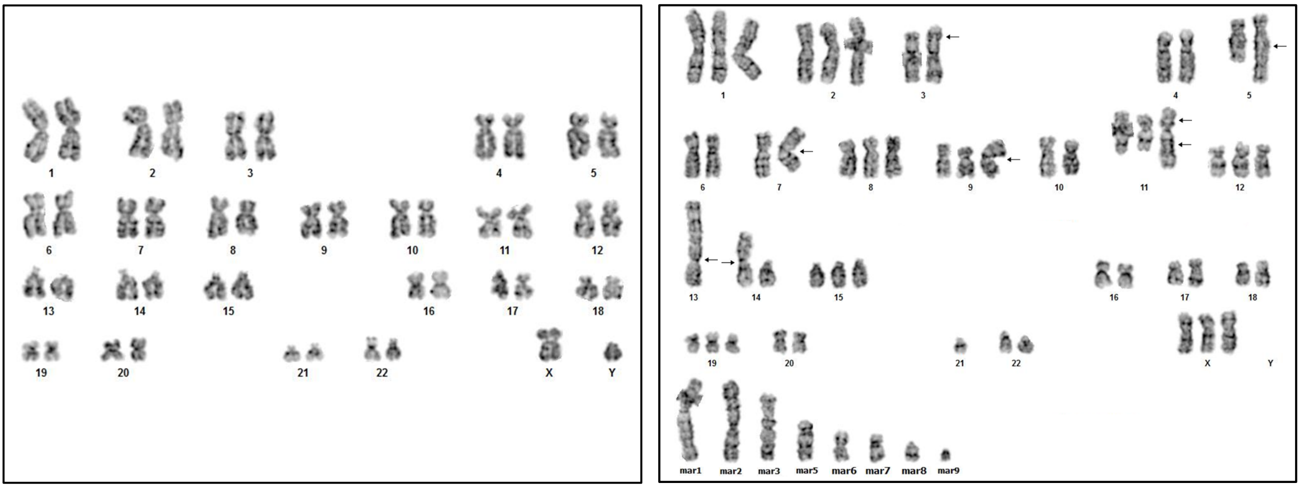

K4DT **SV40T_no serum**

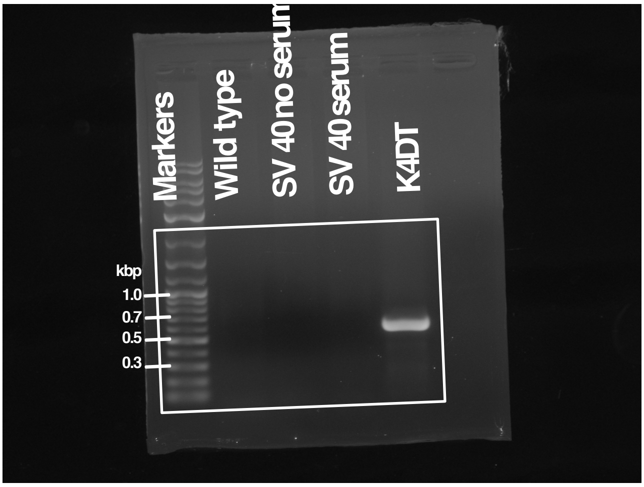

CDK4

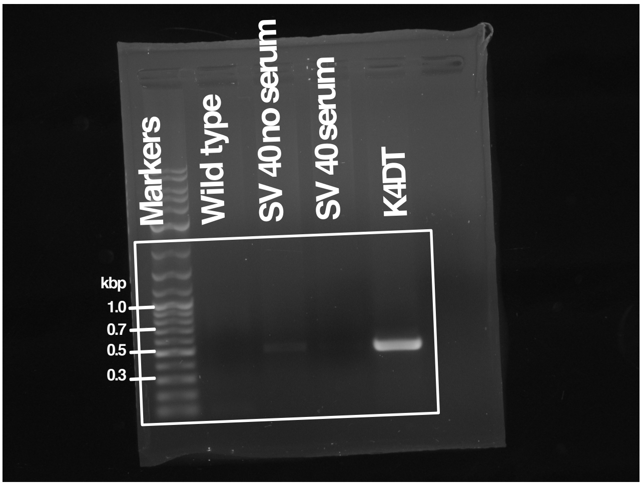

Cyclin D1

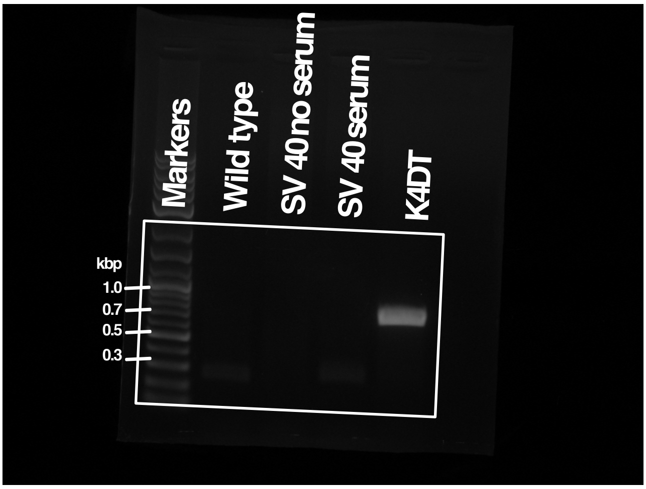

TERT

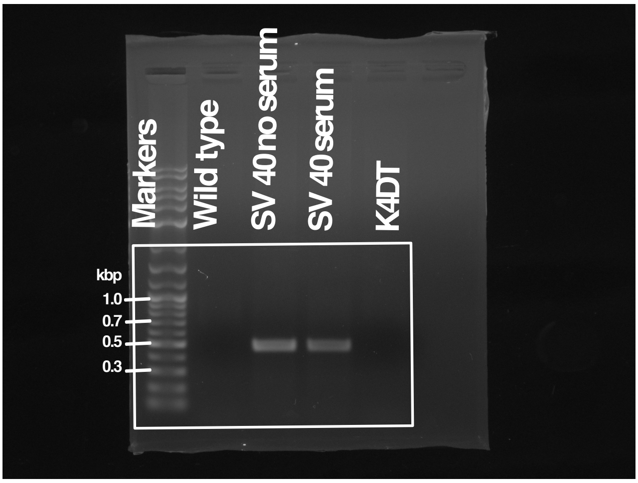

SV40

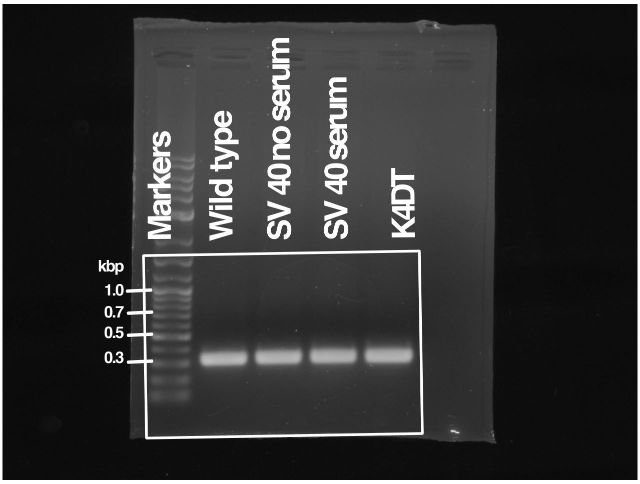

TSC2

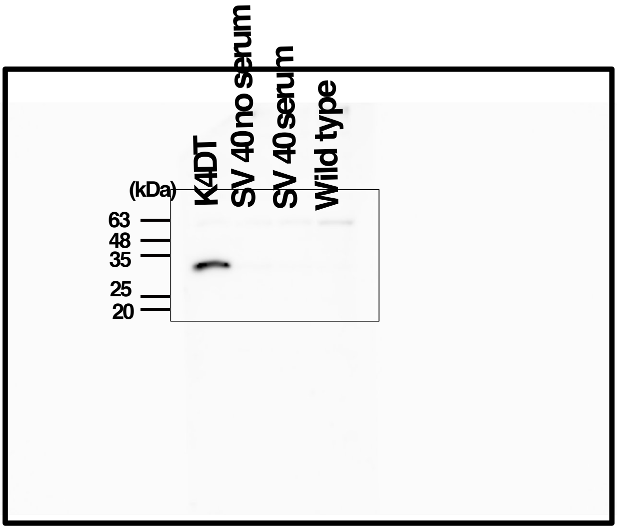

anti-CDK4

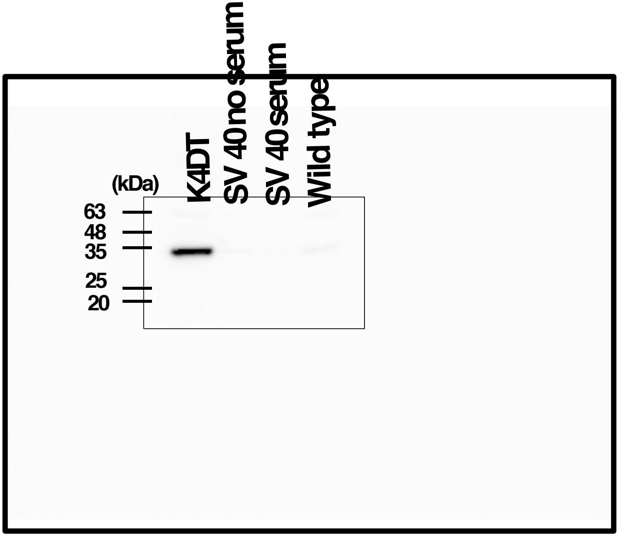

anti-Cyclin D1

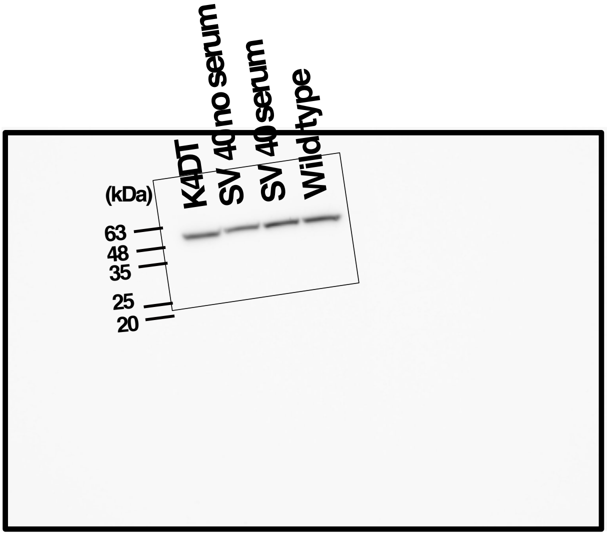

anti-tubulin

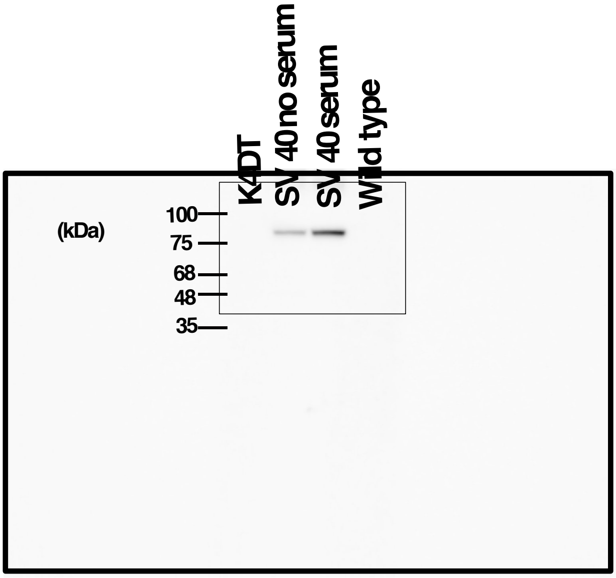

anti-SV40
